# Evaluation of the structural quality of modeled proteins by using globularity criteria

**DOI:** 10.1186/1472-6807-7-9

**Published:** 2007-03-09

**Authors:** Susan Costantini, Angelo M Facchiano, Giovanni Colonna

**Affiliations:** 1 CRISCEB (Research Center of Computational and Biotechnological Sciences), Second University of Naples, Via Costantinopoli 16, 80138 Naples, Italy; 2 Department of Biochemistry and Biophysics, Second University of Naples, Via Costantinopoli 16, 80138 Naples, Italy; 3 Laboratory of Bioinformatics and Computational Biology, Institute of Food Science, CNR, Via Roma 52 A/C, 83100 Avellino, Italy

## Abstract

**Background:**

The knowledge of the three-dimensional structure of globular proteins is fundamental for a detailed investigation of their functional properties. Experimental methods are too slow for structure investigation on a large scale, while computational prediction methods offer alternatives that are continuously being improved. The international Comparative Assessment of Structure Prediction (CASP), an "a posteriori" evaluation of the quality of theoretical models when the experimental structure becomes available, demonstrates that predictions can be successful as well as unsuccessful, and this suggests the necessity for evaluations able to discard "a priori" the wrong models.

**Results:**

We analyzed different structural properties of globular proteins for experimentally solved proteins belonging to the four different structural classes: "mainly alpha", "mainly beta", "alpha/beta" and "alpha+beta". The properties were found to be linearly correlated to protein molecular weight, but with some differences among the four classes. These results were applied to develop an evaluation test of theoretical models based on the expected globular properties of proteins. To verify the success of our test, we applied it to several protein models submitted to the sixth edition of CASP. The best theoretical models, as judged by CASP assessors, were in agreement with the expected properties, while most of the low-quality models had not passed our evaluations.

**Conclusion:**

This study supports the need for careful checks to avoid the diffusion of incorrect structural models. Our test allows the evaluation of models in the absence of experimental reference structures, thereby preventing the diffusion of incorrect structural models and the formulation of incorrect functional hypotheses. It can be used to check the globularity of predicted models, and to supplement other methods already used to evaluate their quality.

## Background

Globular proteins are critical players in the cell whose function is dictated by their characteristic structure. Because the number of proteins with known sequence far exceeds the number with known structure, the ability of computational methods to predict the structure from sequence is considered extremely valuable to investigate their functional properties [1]. Proper valuation of the quality of models is the basic problem of any computational approach to structure prediction. A basic difference between experimental and computational approaches to solve the 3D structure of proteins is that X-ray or NMR protocols start from high protein concentration conditions, while "ab initio" predictive methods run on a single protein molecule. This means that most predictive methods can not take into account the strong influence of environment on the globular structure of the protein. Fundamental features for determining the globularity are solubility, packing stability, folding, and compactness [2,3]. Therefore, to improve the quality of protein structure prediction, their effects should be simulated. As an alternative, at least to evaluate the quality of the predictions, it is necessary to know whether the structural properties typical of globular proteins are retained also by theoretical models.

In 1951 Pauling was the first to consider the importance of the intra-molecular H-bonds in protein structures, emphasizing their role in stabilizing the alpha-helices and beta-strands [4,5]. Many studies have pointed out that H-bonds contribute favorably to globular protein stability [6]. Also important in understanding protein globularity is the packing of the protein atoms (i.e., the efficient filling of space). Protein cavities or packing defects occur with relative abundance, both within and between folding units, and the creation or filling of such cavities affects protein stability and structure, disturbing core packing [7,8]. Moreover, the variation of atom packing in a data set of globular proteins can be due to a complex combination of protein size and secondary structure, and amino acid composition [9]. These differences in protein packing are conserved in protein families of similar structure; the modeling of protein structures based on homologous templates should take into account the packing of the template structure. However, the prediction of protein models obtained by "ab-initio" methods is much more difficult, since there is no template structure as reference. In these cases, and in general for all predicted models, we should always control those parameters able to confirm that the obtained structures have the typical properties of globular proteins. A search of the scientific literature found only two methods that make predictions for globularity, and both are sequence-based rather than structure-based [10,11].

Therefore, we first studied and analyzed a valuable set of experimental protein structures belonging to the four known structural classes ("mainly alpha", "mainly beta", "alpha/beta", and "alpha+beta") in terms of H-bonds, voids, solvent-accessible surface area, and water molecules in a layer of 5 Ångstroms. This analysis allowed us to deduce structural parameters useful in determining the protein globularity and to define operative criteria to evaluate models, particularly those predicted by "ab-initio" methods. These structural parameters were combined together as an index of globularity by which we tested thirteen sets of models submitted to CASP6 protein structure prediction experiment in the New Fold (NF) and difficult Fold Recognition Analogous (FR/A) categories.

## Results

### Protein set selection

Protein structures, solved by NMR or X-Ray crystallography with resolution of 2.5 Å or better, were extracted from PDBselect [12]. These proteins were subdivided into four structural classes, i.e. "mainly alpha", "mainly beta", "alpha/beta" and "alpha+beta", on the bases of secondary structures assigned by DSSP program and of the SCOP database to classify the alpha-beta proteins as alpha+beta or alpha/beta [13-15]. Table 1 reports the number of structures comprised in each set and the mean ratios between the residue number in helix, in beta-strands and in "coil", and the total residue number. These structures were analyzed in terms of H-bonds, accessible surface area, water molecules and voids, in order to define some typical parameters for each protein structural class.

### Hydrogen bonding

**Figure 1:**
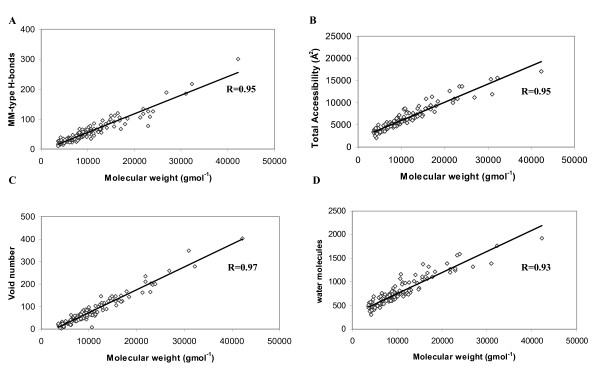
**Parameters plotted against values of molecular weights obtained for each protein belonging to "mainly-alpha" class**. (A) MM-type H-bonds (B) total accessibility (C) void number (D) water molecules. The data were fit by linear least squares (the equations obtained are in Additional file 4). The related correlation coefficients (R) are also reported.

The H-bonds in these protein structures were evaluated using the Hbplus package and classified as main-chain donor to main-chain acceptor (MM), main-chain donor to side-chain acceptor (MS), side-chain donor to main-chain acceptor (SM) and side-chain donor to side-chain acceptor (SS). The total number of H-bonds ranged between 14 and 377 in "mainly-alpha", 9 and 145 in "mainly-beta", 15 and 351 in "alpha+beta" and 41 and 400 in "alpha/beta" proteins. The correlation coefficients between the total number of H-bonds or MM-, MS-, SM- and SS-type H-bonds are reported in Table 2. The total number of MM-type H-bonds increased linearly with the molecular weight of selected proteins belonging to all four protein sets (Figures 1 and Additional Files 1, 2, 3). The mean ratios between MM-, MS-, SM- or SS-type H-bonds and the total number of H-bonds were evaluated and are reported in Table 3A.

### Accessible surface area

For each selected structure the total accessible surface area was evaluated by summing the related polar and non-polar components. The total area accessible to solvent increased in a linear way with the protein molecular weights (Figures 1 and Additional Files 1, 2, 3) with correlation coefficients higher than 0.9 in all four structural classes (Table 2). The mean ratios between non-polar accessibility value and the related total accessibility are reported in Table 3B.

### Water molecules

A layer of 5 Å around each protein was considered, and the number of water molecules and of H-bonds between the water molecules and the protein residues was evaluated. The number of water molecules increased linearly with the molecular weight of the proteins in all four sets with correlation coefficients higher than 0.9 (Table 2, Figures 1 and Additional Files 1, 2, 3).

### Void analysis

The AVP program was run on the four protein sets using two different probes: the first to identify the holes in the interior of a protein (a zero-sized probe) and the second to delimit the solvent accessible regions on the surface (probe with a radius of 1.4). The total void volume is obtained summing the total buried and surface void volumes. This increased linearly with the protein size with correlation coefficients higher than 0.9 (see Table 2, Figures 1 and Additional Files 1, 2, 3), according to Fleming and Richards [9]. The mean ratios between the total surface and buried void volumes and the total void volume are reported in Table 3C.

### Score value for training proteins

By using the corresponding linear regression equations obtained for the structural parameters (see Additional File 4), we computed a score for each protein (see Methods for details). The score value increases if the protein properties are well outside the expected values. For each protein set, we determined the threshold (or cutoff) value as the score which includes 90% of the examined proteins. The threshold value was found to be 5.9 in the case of "mainly-alpha" and "mainly-beta" proteins, and 5.1 for "alpha/beta" and "alpha+beta" proteins. The remaining 10% of analyzed structures presents long amino acid segments in irregular secondary structure at the beginning or end of the chain, which might explain their higher score value. These score values were then similarly computed to evaluate the structural quality of the theoretical protein models.

### Testing protein evaluation

Targets from the CASP6 experiment were selected as a testing set, choosing only full-chain structures (see Methods for selection criteria). The list of the thirteen target models selected is reported in Table 4. These models were analyzed using the procedure reported above and their score values were calculated. Of the total number of models 54.6% (2285) showed scores higher than the threshold value, indicating that a large number of the theoretical models submitted to the CASP competition do not agree with the expected globularity features. It should also be noted that all those models considered to be the best submitted for each target [16,17], as well as the relative experimental structures deposited in PDB, had score values lower than the threshold value (Table 4). This confirms the quality of the best theoretical models submitted to the competition, and likewise confirms the reliability of our evaluation score.

## Discussion

In this work four sets of protein structures were selected from the PDB, classified as "mainly-alpha", "mainly-beta", "alpha+beta" and "alpha/beta" (see Table 1) and analyzed in terms of H-bonds, void number, solvent-accessible surface area and water molecules, comprised in a layer of 5 Ångstroms.

The mean ratios between MM-, MS-, SM- or SS-type H-bonds and the total number of H-bonds, reported in Table 3A, indicated that the MM-type H-bonds were the most frequent in all four structural classes. This agreed with the previous analysis applied to a set of globular proteins, which had found that most H-bonds were local and situated between backbone atoms in proteins, and that almost all were within single elements of the secondary structure [3]. The total number of MM-type H-bonds increased linearly with the molecular weight of selected proteins with correlation coefficients higher than 0.9 in "mainly-alpha", "alpha+beta" and "alpha/beta" proteins (Table 2, Figures 1 and Additional Files 1, 2, 3). In "mainly-beta" proteins the total number of H-bonds was smaller than in other sets, with the correlation coefficient between the MM-type H-bonds and the protein molecular weights found to be 0.83. This smaller value could depend on the higher content of irregular secondary structure in "mainly-beta" proteins (see Table 1).

The total accessible surface area (ASA), with its polar and non-polar components, was evaluated for all proteins of the four structural classes. The correlation coefficients between the non-polar components and the molecular weights were found to be higher than those obtained for the polar components in "mainly-alpha", "mainly-beta" and "alpha/beta" proteins, but not in "alpha+beta" proteins. In fact, in these structures the non-polar component was slightly higher than the polar one, as indicated by the mean ratios between non-polar accessibility values and the relative total accessibility (Table 3B).

Moreover, as for the 5 Å layer around each protein, we verified that the number of water molecules increased linearly with the molecular weight of the proteins in all four sets (Figures 1 and Additional Files 1, 2, 3). The correlation coefficients, calculated for proteins belonging to all four classes, between water-protein H-bonds and the relative total accessibility values were found to be consistently higher than those between water-protein H-bonds and the protein molecular weights (Table 2). This suggests that both protein shape and surface extension may have a greater effect on the number of H-bond interactions between proteins and water than does protein size.

Finally, the voids were analyzed in all proteins to assess both packing quality and individual voids [18,19]. The correlation coefficient between the largest void volumes and the total void volumes, calculated for all proteins belonging to each given class, was 0.76 in "mainly-alpha", 0.71 in "mainly-beta", 0.71 in "alpha+beta" and 0.58 in "alpha/beta" proteins, respectively. These values indicated that the total void volume of each structure belonging to "mainly-alpha", "mainly-beta" and "alpha+beta" classes represents mainly the largest void present, in agreement with results obtained analyzing a heterogeneous dataset [18]. The mean ratios between the total surface and buried void volumes and the total void volume (Table 3C) indicated that the selected structures had a greater number of voids on the surface and few buried voids, and that the total void volume consisted mainly of the surface void volume. The presence of few buried voids was used to verify that the selected proteins are well-packed and compact [7,20]. Moreover, the total void volumes are <1500 Å ^3 ^in "mainly-alpha" and "mainly-beta" proteins, and >3000 Å ^3 ^in those "alpha/beta" and "alpha+beta", in which more buried voids are also present (see Table 3C). These values could indicate that the "mainly-alpha" and "mainly-beta" proteins are more compact than the others.

As a final comment on the evaluation of these globularity features, we note that some differences were seen among the four structural classes, whose cause requires further investigation. Different degrees of compactness as well as flexibility may be needed depending on the structural architecture which is often related to the functional role. As an example, the multi domain nature of alpha+beta proteins is often related to the need to open the protein globe and offer a larger cavity to fit a ligand. Likewise, as in the case of barrel architecture, alpha/beta proteins show a large internal cavity which could explain the differences in void ratios for this class. However, other hypotheses could be postulated and this aspect of the results warrants further investigation.

**Figure 2:**
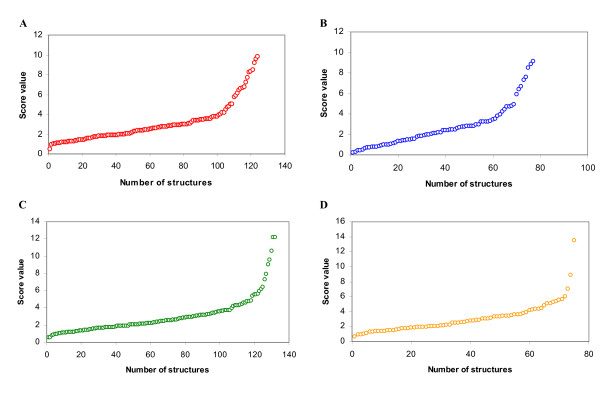
**Score value calculated for all the proteins belonging to the four structural classes**. (A) "mainly-alpha", (B) "mainly-beta", (C) "alpha+beta" and (D) "alpha/beta". The score values are calculated summing for all the proteins the ratios of the differences between the calculated and predicted values for each of four properties (number of MM-type H-bonds, void and water molecules, and total accessibility) versus the related RMSE.

Our analyses have shown that some globular properties are well conserved in proteins within the same structural class. Therefore, from these studies we obtained some theoretical parameters, i.e. linear regression equations and Root Mean Squared Error (RMSE), shown in Additional File 4, specific for each structural class. These parameters could be useful in evaluating models, especially those predicted by "ab-initio" methods, for which reference structures are not available. A score value for all proteins was calculated by using the parameters having the highest correlation coefficients with the protein molecular weights (MM-type H-bonds, void number, total accessible surface area and water molecules) (see Figure 2).

To test the applicability and usefulness of these criteria, thirteen targets of CASP experiment were selected. Only the full-atom structures were chosen for each testing set [16,17]. Our results surprisingly show that many of the models submitted (54.6%) should be discarded *a priori *because they do not have the structural properties expected in globular proteins.

An interesting aspect concerns the subdivision of prediction methods present in CASP6 as "human" and "server" predictors [21]. The results shown in Table 5 separate the two classes of predictors. The models exceeding the threshold value of our globularity score were 51.6% and 64.2% for human and server predictors, respectively.

The threshold value has been applied in our analysis as a cutoff which creates two subsets of models, the one below the cutoff should include models with globularity features in agreement with those expected in crystallographic structures, while the subset of models above the cutoff should include models with poor globularity features. To validate this, we evaluated the average model quality for the two subsets by using root-mean square deviations (RMSD) and Global Distance Test Total Score (GDT_TS) reported in CASP6 tables, as well as MaxSub score [22], by considering these parameters as correct model quality measures.

For each target and for the whole set, the average values of RMSD and GDT_TS were evaluated for the two subsets of models (see Additional File 5). The subset of the models below the cutoff resulted always (i.e., in the whole set as well as for each target) to have the best average quality, compared to the other subset.

Moreover, we correlated our globularity score with a number of model evaluation parameters, i.e. gross violations of distance constraints (err), RMSD and GDT_TS reported in CASP6 tables, as well as MaxSub score. In addition, we applied to the models other quality assessment programs, i.e. ProsaII Z-score [23], Modcheck score [24], Anolea Z-score [25,26], and Victor/FRST function [27], and compared these scores with our globularity score. The plots for each comparison are shown in Additional Files 6, 7, 8, 9, 10, 11, 12, 13, 14, 15, 16, 17, 18, 19, corresponding to each of thirteen targets analyzed and to the whole set. In particular, 79% of models with globularity scores lower than the threshold values have less than 3 distance violation (err). Overall, the trend indicates that the threshold value of the globularity score selects the better models as evaluated by the different parameters. The linear correlation coefficients suggest that the globularity score is not directly related to ProsaII Z-score, Modcheck score, Anolea Z-score, and Victor/FRST function. This suggests that our globularity score cannot simply be derived from other evaluation parameters, which means that our score can be used as an independent evaluation criterion.

The correlation coefficients between our globularity score, as well as all quality assessment programs listed above, and the correct quality measures (i.e. RMSD, GDT_TS and MaxSub) were evaluated for the whole set (see Table 6) and for thirteen targets (see Additional File 20). The globularity score has the better correlation with RMSD and GDT_TS.

In Tables 6 and Additional File 20 we have also reported the correlation coefficients between the four individual features (MM-type H-bonds, void number, water molecules and total accessibility) used in our work and the correct measures (RMSD, GDT_TS and MaxSub).

The combination of the four parameters in our score offer certainly some advantage. In fact, we have also evaluated the number of models in every dataset having the void number, MM-type H-bonds, water molecules and total accessibility in the ranges defined by the predicted values ± RMSE (see Additional Files 4 and 21-A). These results showed that the four structural properties are independent. The average GDT_TS measures for the models which pass the selection with the single features were also evaluated in order to know their quality (see Additional File 21-B). The average GDT_TS values for the whole set of selected models range from 22.4 to 22.9. By considering that the average GDT_TS for the subsets below and above the cutoff (see Additional File 5) were 24.1 and 15.2, respectively, we observe that the subset with the better quality is that selected by the globularity score. The exclusion of models by considering the simple range of each parameter, or the combination of two, three, or four parameters (see Additional File 22) may be too restrictive and in some cases would even disqualify the best theoretical models submitted for the targets. On the contrary, the use of the globularity score and the related value allows some models to pass the threshold even when some of four parameters do not fall within the range.

Moreover, all the experimental structures, deposited in PDB, and the theoretical models indicated as the best submitted for these targets [16,17], had score values below the thresholds (≤ 5.1 or ≤ 5.9, depending on the structural class). This confirms that our method provides reliable results. It should be noted that globularity is a spread property of proteins, but some proteins may have poor globularity features. Therefore, we strongly suggest using our scoring method only for proteins expected to be globular. Finally, our study suggests that the evaluation of theoretical models can be improved by taking into account the globularity features before releasing the models, submitting them to CASP, or using them for further studies.

## Conclusion

We have analyzed structural properties that characterize protein globularity and have suggested an operative procedure to be used for the analysis of globular quality of theoretical protein models, obtained by computational approaches in the absence of experimental target structures. Our scoring method is a tool to avoid the diffusion of incorrect structures and of incorrect functional hypotheses, that can be employed to check the globularity of predicted models and to supplement other methods already used to evaluate their quality [28,29].

## Methods

### Training datasets and their analysis

The list of protein structures used in this study was obtained using PDBselect, a set of experimentally determined, non-redundant protein structures in the PDB. The secondary structure for each PDB entry was assigned by the DSSP algorithm based on the analysis of backbone dihedral angles and hydrogen bonds. DSSP assigns seven different secondary structures, i.e., H: alpha-helix, G: 3_10 _helix, I: π-helix, E: extended strand, B: residue in isolated beta-bridge, S: bend, and T: H-bonded turn. In addition, a "coil" state is assigned when no secondary structure is recognized [13]. We applied the convention to define H, G, I as helix, E and B as strand, and others as coil [30].

A Perl script was written to select the monomeric protein structures, that contain only alpha-helices ("mainly-alpha"), only beta- strands ("mainly-beta") and alpha-helices and beta- strands ("alpha-beta") on the basis of the clearly defined classification [31]. The "alpha-beta" proteins were subdivided in "alpha+beta" and "alpha/beta" on the basis of SCOP classification [14,15]. For the structures determined by NMR, only one chain in the file was considered.

Hydrogen atoms were added to the protein structures with the "Modify/Add Hydrogens tool" in InsightII package (Accelrys, Inc., San Diego, CA). The Hbplus package was used to evaluate the putative formation of H-bonds. It identifies H-bonds within a distance of 2.5 Ångstroms and a minimum angle of 90° [32]. Solvent accessibility of amino acids was evaluated by the NACCESS program, calculating the atomic accessible surface defined by rolling a probe of 1.40 Ångstroms around the van der Waals surface of every protein structure [33]. The AVP program was used to analyze voids in proteins, defined as empty cavities not accessible to solvent [18]. It uses a simple grid-based method and separates the probe size used to define voids from the probe size used to define channels to the surface. This method combines the analysis of individual discrete voids with that of packing quality and can be applied to the calculation of total void volume, maximum void size, and number of voids.

A layer of water molecules around every protein was added using the tool "Assembly→Soak→Layer" in InsightII. The H-bonds between the residue atoms in protein and the water molecules was evaluated by Hbplus program.

Further Perl scripts were written to apply the AVP, Hbplus and NACCESS programs to the selected protein dataset.

### Linear regression and RMSE

The total accessible surface area (ASA), the number of MM-type H-bonds, voids and water molecules versus the molecular weights of selected proteins in each structural class fit to linear regressions described by four equations relating the *y *values (i.e. *y*_*ASA*_, *y*_*MM*_, *y*_*void *_and *y*_*water*_) to the *x *values (i.e. molecular weights). For each of four properties the Root Mean Squared Error (*RMSE*) was calculated, that is the average distance of all the points from the fitted line, measured along a vertical line

RMSE=EiC=1NC∑i=1N(yi−yi')2
 MathType@MTEF@5@5@+=feaafiart1ev1aaatCvAUfKttLearuWrP9MDH5MBPbIqV92AaeXatLxBI9gBaebbnrfifHhDYfgasaacH8akY=wiFfYdH8Gipec8Eeeu0xXdbba9frFj0=OqFfea0dXdd9vqai=hGuQ8kuc9pgc9s8qqaq=dirpe0xb9q8qiLsFr0=vr0=vr0dc8meaabaqaciaacaGaaeqabaqabeGadaaakeaacqWGsbGucqWGnbqtcqWGtbWucqWGfbqrcqGH9aqpcqWGfbqrdaqhaaWcbaGaemyAaKgabaGaem4qameaaOGaeyypa0ZaaOaaaeaadaWcaaqaaiabigdaXaqaaiabd6eaonaaCaaaleqabaGaem4qameaaaaakmaaqahabaWaaeWaaeaacqWG5bqEdaWgaaWcbaGaemyAaKgabeaakiabgkHiTiabdMha5naaDaaaleaacqWGPbqAaeaacqGGNaWjaaaakiaawIcacaGLPaaadaahaaWcbeqaaiabikdaYaaaaeaacqWGPbqAcqGH9aqpcqaIXaqmaeaacqWGobGta0GaeyyeIuoaaSqabaaaaa@4BB5@

where *N*_*C *_is the total number of structures in each class, *y*_*i *_is the value predicted by linear equation in each of the four cases (i.e. *y*_*ASA*_, *y*_*MM*_, *y*_*void *_and *y*_*water*_) and *y'*_*i *_is the corresponding calculated value.

### Score value

A score value was calculated for all the proteins, belonging to one class, by summing the ratios of the differences between the calculated and predicted values for each of the four properties versus the related errors (*E*_*i*_^*C*^).

σC=[|yASA−yASA'|EASAC+|yMM−yMM'|EMMC+|yvoid−yvoid'|EvoidC+|ywater−ywater'|EwaterC]
 MathType@MTEF@5@5@+=feaafiart1ev1aaatCvAUfKttLearuWrP9MDH5MBPbIqV92AaeXatLxBI9gBaebbnrfifHhDYfgasaacH8akY=wiFfYdH8Gipec8Eeeu0xXdbba9frFj0=OqFfea0dXdd9vqai=hGuQ8kuc9pgc9s8qqaq=dirpe0xb9q8qiLsFr0=vr0=vr0dc8meaabaqaciaacaGaaeqabaqabeGadaaakeaaiiGacqWFdpWCdaahaaWcbeqaaiabdoeadbaakiabg2da9maadmaabaWaaSaaaeaadaabdaqaaiabdMha5naaBaaaleaacqWGbbqqcqWGtbWucqWGbbqqaeqaaOGaeyOeI0IaemyEaK3aa0baaSqaaiabdgeabjabdofatjabdgeabbqaaiabcEcaNaaaaOGaay5bSlaawIa7aaqaaiabdweafnaaDaaaleaacqWGbbqqcqWGtbWucqWGbbqqaeaacqWGdbWqaaaaaOGaey4kaSYaaSaaaeaadaabdaqaaiabdMha5naaBaaaleaacqWGnbqtcqWGnbqtaeqaaOGaeyOeI0IaemyEaK3aa0baaSqaaiabd2eanjabd2eanbqaaiabcEcaNaaaaOGaay5bSlaawIa7aaqaaiabdweafnaaDaaaleaacqWGnbqtcqWGnbqtaeaacqWGdbWqaaaaaOGaey4kaSYaaSaaaeaadaabdaqaaiabdMha5naaBaaaleaacqWG2bGDcqWGVbWBcqWGPbqAcqWGKbazaeqaaOGaeyOeI0IaemyEaK3aa0baaSqaaiabdAha2jabd+gaVjabdMgaPjabdsgaKbqaaiabcEcaNaaaaOGaay5bSlaawIa7aaqaaiabdweafnaaDaaaleaacqWG2bGDcqWGVbWBcqWGPbqAcqWGKbazaeaacqWGdbWqaaaaaOGaey4kaSYaaSaaaeaadaabdaqaaiabdMha5naaBaaaleaacqWG3bWDcqWGHbqycqWG0baDcqWGLbqzcqWGYbGCaeqaaOGaeyOeI0IaemyEaK3aa0baaSqaaiabdEha3jabdggaHjabdsha0jabdwgaLjabdkhaYbqaaiabcEcaNaaaaOGaay5bSlaawIa7aaqaaiabdweafnaaDaaaleaacqWG3bWDcqWGHbqycqWG0baDcqWGLbqzcqWGYbGCaeaacqWGdbWqaaaaaaGccaGLBbGaayzxaaaaaa@9630@

As for the most frequent score values, calculated for the proteins belonging to the same class, it was possible to identify a score value specific for each of the four structural classes. These score values were then used to evaluate the structural properties of models predicted by folding "ab-initio" methods and used as "testing dataset".

### Testing protein dataset

The testing structures were selected starting from all the protein models predicted for the CASP6 protein structure prediction experiment in the New Fold (NF) and difficult Fold Recognition Analogous (FR/A) categories [16,17]. To analyze globularity features, we selected only those models for which complete chains are available, for both the experimental and the theoretical structures. Following these criteria thirteen targets were selected, i.e. T0198 (PDB code: 1SUM (3–223)) and T0238 (1W33 (70–222)) classified as "mainly-alpha", T0212 (1TZA (3–121)) and T0209_1 (1XQB (9–138)) as "mainly-beta", T0199_3 (1STZ (145–226)), T0201 (1S12 (1–90)), T0209_2 (1XQB(159–221), T0216_1 (1VL4(2–214)), T0216_2 (1VL4(221–433)), T0239 (1RKI(1–98)) and T0248_2 (1TD6 (107–193)) as "alpha+beta" and T0242 (2BLK (2–116)) and T0273 (1WDJ(2–187)) as "alpha/beta". All theoretical models submitted for each of these targets were used as testing structures, but only if the full atom model was available. These models were analyzed with the same software used for the training protein structures and their structural properties were evaluated by the score values.

Next, our globularity scores were compared with some model evaluation parameters, i. e. gross violations of distance constraints (err), root-mean square deviations (RMSD) and Global Distance Test_Total Score (GDT_TS) reported in CASP6 tables.

Finally, all models were analyzed also by MaxSub program [22], PROSA II Z-score [23], Modcheck score [24], Anolea Z-score [25,26], and Victor/FRST function [27], using the default parameters for each of these. Briefly, MaxSub measures the similarity of a model to its corresponding experimental structure reporting 0 for a completely wrong model and 1 for a perfect model. Prosa II and Anolea compute a Z-score value indicating the better model on the basis of the lower value but Modcheck on the basis of the higher value. Victor/FRST package calculates a pseudo-energy to evaluate the quality of a given protein structural model for which the lower score reflects the better model.

### Web site

A web site has been devoted to the additional materials we produced during this work and to add in the time more evaluations. The site is freely accessible [34].

## Authors' contributions

AMF and GC conceived of the study and participated in its design and coordination. SC carried out the computational experiments and analysis of the results. All authors read and approved the final manuscript.

## Supplementary Material

Additional File 1 **Figure1S**. Parameters plotted against values of molecular weights obtained for each protein, belonging to "mainly-beta" class. (A) MM-type H-bonds (B) total accessibility (C) void number (D) water molecules. Click here for file

Additional File 2 **Figure2S**. Some parameters plotted against values of molecular weights obtained for each protein, belonging to "alpha/beta" class. (A) MM-type H-bonds (B) total accessibility (C) void number (D) water molecules. Click here for file

Additional File 3 **Figure3S**. Some parameters plotted against values of molecular weights obtained for each protein, belonging to "alpha+beta" class. (A) MM-type H-bonds (B) total accessibility (C) void number (D) water molecules. Click here for file

Additional File 4 **Table1S**. Theoretical parameters obtained analysing protein structures belonging to four structural classes. Click here for file

Additional File 5 **Table2S**. We have reported for each target and for the whole model set the cutoff value of globularity score, and the average values of RMSD and GDT_TS for the models above and below the cutoff. In parenthesis are reported the standard deviations. Click here for file

Additional File 6 **Figure4S**. Model evaluation parameters plotted against values of globularity score obtained for T0198 target (A) gross violations of distance constraints (err) (B) root-mean square deviations (RMSD) (C) Global Distance Test_Total Score (GDT_TS) (D) MaxSub score (E) PROSA II Z-score (F) Victor/FRST function (G) Modcheck score (H) Anolea Z-score. Click here for file

Additional File 7 **Figure5S**. Model evaluation parameters plotted against values of globularity score obtained for T0201 target (A) gross violations of distance constraints (err) (B) root-mean square deviations (RMSD) (C) Global Distance Test_Total Score (GDT_TS) (D) MaxSub score (E) PROSA II Z-score (F) Victor/FRST function (G) Modcheck score (H) Anolea Z-score. Click here for file

Additional File 8 **Figure6S**. Model evaluation parameters plotted against values of globularity score obtained for T0212 target (A) gross violations of distance constraints (err) (B) root-mean square deviations (RMSD) (C) Global Distance Test_Total Score (GDT_TS) (D) MaxSub score (E) PROSA II Z-score (F) Victor/FRST function (G) Modcheck score (H) Anolea Z-score. Click here for file

Additional File 9 **Figure7S**. Model evaluation parameters plotted against values of globularity score obtained for T0238 target (A) gross violations of distance constraints (err) (B) root-mean square deviations (RMSD) (C) Global Distance Test_Total Score (GDT_TS) (D) MaxSub score (E) PROSA II Z-score (F) Victor/FRST function (G) Modcheck score (H) Anolea Z-score. Click here for file

Additional File 10 **Figure8S**. Model evaluation parameters plotted against values of globularity score obtained for T0242 target (A) gross violations of distance constraints (err) (B) root-mean square deviations (RMSD) (C) Global Distance Test_Total Score (GDT_TS) (D) MaxSub score (E) PROSA II Z-score (F) Victor/FRST function (G) Modcheck score (H) Anolea Z-score. Click here for file

Additional File 11 **Figure9S**. Model evaluation parameters plotted against values of globularity score obtained for T0248 target (A) gross violations of distance constraints (err) (B) root-mean square deviations (RMSD) (C) Global Distance Test_Total Score (GDT_TS) (D) MaxSub score (E) PROSA II Z-score (F) Victor/FRST function (G) Modcheck score (H) Anolea Z-score. Click here for file

Additional File 12 **Figure10S**. Model evaluation parameters plotted against values of globularity score obtained for T0216_1 target (A) gross violations of distance constraints (err) (B) root-mean square deviations (RMSD) (C) Global Distance Test_Total Score (GDT_TS) (D) MaxSub score (E) PROSA II Z-score (F) Victor/FRST function (G) Modcheck score (H) Anolea Z-score. Click here for file

Additional File 13 **Figure11S**. Model evaluation parameters plotted against values of globularity score obtained for T0216_2 target (A) gross violations of distance constraints (err) (B) root-mean square deviations (RMSD) (C) Global Distance Test_Total Score (GDT_TS) (D) MaxSub score (E) PROSA II Z-score (F) Victor/FRST function (G) Modcheck score (H) Anolea Z-score. Click here for file

Additional File 14 **Figure12S**. Model evaluation parameters plotted against values of globularity score obtained for T0239 target (A) gross violations of distance constraints (err) (B) root-mean square deviations (RMSD) (C) Global Distance Test_Total Score (GDT_TS) (D) MaxSub score (E) PROSA II Z-score (F) Victor/FRST function (G) Modcheck score (H) Anolea Z-score. Click here for file

Additional File 15 **Figure13S**. Model evaluation parameters plotted against values of globularity score obtained for T0199_3 target (A) gross violations of distance constraints (err) (B) root-mean square deviations (RMSD) (C) Global Distance Test_Total Score (GDT_TS) (D) MaxSub score (E) PROSA II Z-score (F) Victor/FRST function (G) Modcheck score (H) Anolea Z-score. Click here for file

Additional File 16 **Figure14S**. Model evaluation parameters plotted against values of globularity score obtained for T0209_1 target (A) gross violations of distance constraints (err) (B) root-mean square deviations (RMSD) (C) Global Distance Test_Total Score (GDT_TS) (D) MaxSub score (E) PROSA II Z-score (F) Victor/FRST function (G) Modcheck score (H) Anolea Z-score. Click here for file

Additional File 17 **Figure15S**. Model evaluation parameters plotted against values of globularity score obtained for T0209_2 target (A) gross violations of distance constraints (err) (B) root-mean square deviations (RMSD) (C) Global Distance Test_Total Score (GDT_TS) (D) MaxSub score (E) PROSA II Z-score (F) Victor/FRST function (G) Modcheck score (H) Anolea Z-score. Click here for file

Additional File 18 **Figure16S**. Model evaluation parameters plotted against values of globularity score obtained for T0273 target (A) gross violations of distance constraints (err) (B) root-mean square deviations (RMSD) (C) Global Distance Test_Total Score (GDT_TS) (D) MaxSub score (E) PROSA II Z-score (F) Victor/FRST function (G) Modcheck score (H) Anolea Z-score. Click here for file

Additional File 19 **Figure17S**. Model evaluation parameters plotted against values of globularity score obtained for all models (A) gross violations of distance constraints (err) (B) root-mean square deviations (RMSD) (C) Global Distance Test_Total Score (GDT_TS) (D) MaxSub score (E) PROSA II Z-score (F) Victor/FRST function (G) Modcheck score (H) Anolea Z-score. Click here for file

Additional File 20 **Table3S**. Correlation coefficients between all methods, as well as for our four individual features (MM-type H-bonds, void number, water molecules and total accessibility), and three correct quality measures (i.e. RMSD, GDT_TS and MaxSub) evaluated for each of 13 target. Click here for file

Additional File 21 **Table4S**. A. For each target, columns report the number of models analyzed and the ratios of models for which the voids, MM-type H-bonds, water molecules, the total accessibility and the score values resulted within expected ranges for globular proteins and calculated as reported in Methods (see Additional File 4). B. For each target and for the whole set, columns report average GDT_TS value evaluated for models that have the number of void, MM-type H-bonds and water molecules, and the total accessibility within the expected range. In parenthesis are reported the standard deviations. Click here for file

Additional File 22 **Table5S**. Number of models for which the combination of two, three or four parameters (voids, MM-type H-bonds, water molecules and total accessibility) resulted in the expected ranges for globular proteins and calculated as reported in Methods (see Additional File 4). Click here for file

## Figures and Tables

**Table 1:** Training protein sets.

	**Number of structures**	**helix/total**	**beta/total**	**Coil/total**
**mainly-alpha**	124	0.648(± 0.12)		0.36(± 0.12)
**mainly-beta**	81		0.37(± 0.13)	0.63(± 0.13)
**alpha/beta**	75	0.38(± 0.08)	0.21(± 0.04)	0.41(± 0.07)
**alpha+beta**	132	0.29(± 0.08)	0.27(± 0.079	0.44(± 0.09)

We report the number of structures in the four sets and the mean ratios between the residues in helix, in beta-strands and in "coil" and the total residue number. Standard deviations are reported in parentheses.

**Table 2:** Correlation coefficients in the four structural classes.

	**mainly-alpha**	**mainly-beta**	**alpha/beta**	**alpha+beta**
**H-bonds**				
MM-type	0.95	0.83	0.97	0.96
MS-type	0.61	0.49	0.83	0.79
SM-type	0.70	0.39	0.85	0.81
SS-type	0.67	0.41	0.82	0.81
Total	0.93	0.80	0.97	0.95
				
**Total Accessibility**				
polar-	0.89	0.89	0.93	0.94
non polar-	0.92	0.90	0.95	0.92
Total	0.95	0.93	0.96	0.94
				
**Void**				
total void volumes	0.87	0.9	0.92	0.91
void numbers	0.97	0.91	0.99	0.97
				
**water**				
molecule number	0.93	0.91	0.97	0.93
protein-water H-bonds	0.8	0.76	0.71	0.73
				
protein-water H-bonds/total accessibility	0.84	0.81	0.78	0.73

The correlation coefficients are evaluated between the molecular weights and the different types of H-bonds, the total solvent accessibility and its components, the number of water molecules and the related H-bonds between residue proteins and water molecules, and void number and total void volumes (calculated with probe = 0) obtained for each protein belonging to "mainly-alpha, "mainly-beta", "alpha/beta" and "alpha+beta" classes. The last row reports the correlation coefficient between the total accessibility and the number of H-bonds between residue proteins and water molecules.

**Table 3:** Mean ratios between the components and related total values for some properties.

**A**	**mainly-alpha**	**mainly-beta**	**alpha/beta**	**alpha+beta**
**MM-type/total**	0.84(± 0.10)	0.74(± 0.14)	0.74(± 0.08)	0.77(± 0.11)
**MS-type/total**	0.04(± 0.03)	0.09(± 0.07)	0.08(± 0.03)	0.08(± 0.04)
**SM-type/total**	0.07(± 0.05)	0.10(± 0.07)	0.09(± 0.04)	0.08(± 0.05)
**SS-type/total**	0.05(± 0.05)	0.07(± 0.07)	0.09(± 0.04)	0.07(± 0.05)
**B**				
**non-polar/total**	0.59(± 0.06)	0.57(± 0.05)	0.57(± 0.03)	0.56(± 0.04)
**polar/total**	0.41(± 0.06)	0.43(± 0.05)	0.43(± 0.03)	0.44(± 0.04)
**C**				
**surface/total void volume**	(0.95± 0.05)	0.96(± 0.06)	0.85(± 0.07)	0.92(± 0.07)
**buried/total void volume**	0.05(± 0.04)	0.04(± 0.03)	0.15(± 0.07)	0.08(± 0.06)
**surface/total void number**	0.91(± 0.08)	0.93(± 0.07)	0.76(± 0.08)	0.86(± 0.08)
**buried/total void number**	0.09(± 0.08)	0.07(± 0.07)	0.24(± 0.08)	0.14(± 0.08)

(A) H-bonds, (B) accessible surface area, (c) void. Standard deviations are reported in parentheses.

**Table 4:** Target proteins used as testing dataset.

**Target**	**Class**	**PDB code**	**Score value for PDB structures**	**Number of models**	**Percentage of models with score ≥ 5.9 or ≥ 5.1**
**T0198**	mainly-alpha	1SUM_3-223	5.1	154	90.9
**T0238**	mainly-alpha	1W33_70-222	5.8	154	86.4
**T0209_1**	mainly-beta	1XQB_9-138	3.11	152	87.6
**T0212**	mainly-beta	1TZA_3-121	3.4	182	41.2
**T0199_3**	alpha+beta	1STZ_145-226	2.10	223	50.7
**T0201**	alpha+beta	1S12_1-90	1.6	204	20.6
**T0209_2**	alpha+beta	1XQB_159-231	2.35	229	41
**T0216_1**	alpha+beta	1VL4_2-214	4.3	155	93.5
**T0216_2**	alpha+beta	1VL4_221-433	3.3	147	95.3
**T0239**	alpha+beta	1RKI_1-98	1.8	251	29.1
**T0248_2**	alpha+beta	1TD6_107-193	2.9	239	32.6
**T0242**	alpha/beta	2BLK_2-116	3.1	200	43
**T0273**	alpha/beta	1WDJ_2-187	1.2	141	90

The columns report the target codes, structural class, code of the experimental structure deposited in the PDB with the segment analyzed, their score value, the number of full-atom models analyzed in this work, and the percentage of models for which the score value resulted over the threshold (i.e. ≥ 5.9 for "mainly-alpha" and "mainly-beta" proteins and ≥ 5.1 for "alpha/beta" and "alpha+beta" proteins).

**Table 5:** Details for the analysis of testing datasets.

	**Total number Models**			**Model within expected score**		
	**Total**	**Human**	**Server**	**Total**	**Human**	**Server**

**T0198**	152	110/152	42/152	14/152	9/110	5/42
**T0238**	154	117/154	37/154	21/154	18/117	3/37
**T0209_1**	152	108/152	43/152	20/152	20/108	0/43
**T0212**	181	135/181	46/181	107/181	87/135	20/46
**T0199_3**	223	177/223	46/223	110/223	96/177	14/46
**T0201**	203	165/203	38/203	162/203	136/165	26/38
**T0209_2**	229	172/229	57/229	135/229	103/172	32/57
**T0216_1**	155	123/155	32/155	10/155	6/123	4/32
**T0216_2**	147	114/147	33/147	7/147	6/114	1/33
**T0239**	251	207/251	44/251	178/251	150/251	28/44
**T0248_2**	238	180/238	58/238	161/238	125/180	36/58
**T0242**	200	154/200	46/200	114/200	96/154	18/46
**T0273**	141	107/141	34/141	14/141	10/107	4/34

For each target, columns report the total number of models analyzed, the ratio of models from "human" and "server" predictors, the ratios of models which have globularity score below the threshold value.

**Table 6:** Correlation coefficients for whole set of models.

	**RMS**	**GDT_TS**	**MaxSub**
**Z-score [Prosa]**	0.16	0.04	-0.026
**Modcheck score**	-0.18	-0.11	-0.05
**Victor/FRST function**	0.16	-0.35	-0.27
**Z-score [Anolea]**	0.16	-0.04	-0.14
**Globularity score**	0.56	-0.4	-0.074
**MM-type H-bonds**	0.25	-0.12	0.37
**Void number**	-0.17	0.036	-0.093
**Total accessibility**	0.51	-0.40	0.0066
**Water molecules**	0.54	-0.36	0.077

In columns we report the correlation coefficients between the quality assessment methods (Prosa, Modcheck, Victor/FRST, Anolea and Globularity score), as well as our four individual features (MM-type H-bonds, void number, water molecules and total accessibility), and three correct quality measures (i.e. RMSD, GDT_TS and MaxSub)
