# Supplementary figures and images for: Evaluation of the structural quality of modeled proteins by using globularity criteria
Source: BMC Struct Biol. 2007 Mar 9;7:9. doi: 10.1186/1472-6807-7-9 (PMC1828058; doi:10.1186/1472-6807-7-9)

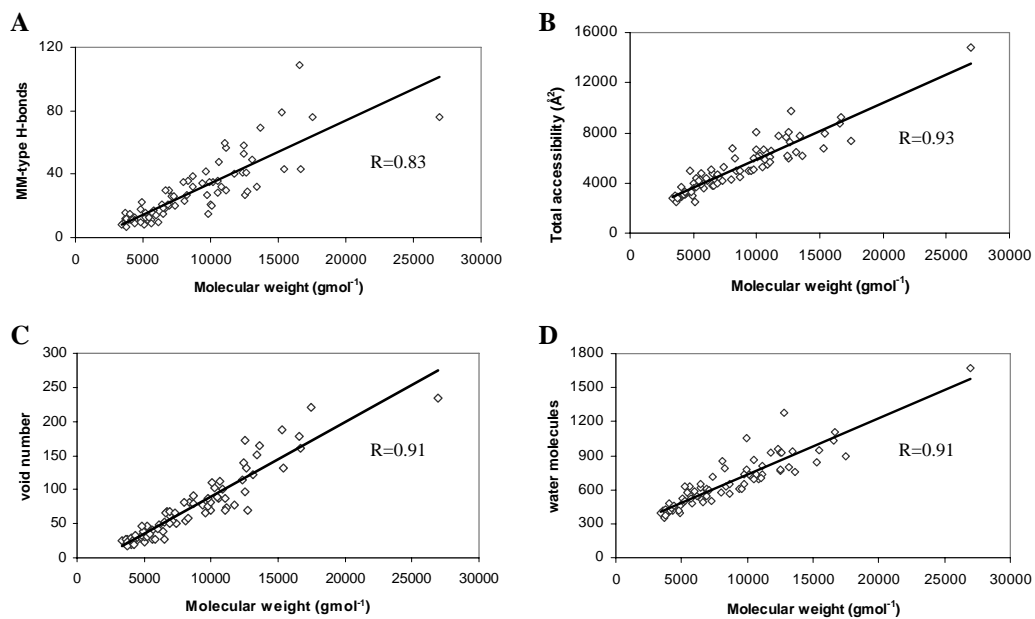

**Figure1S**

Supplement: Additional File 1 — Figure1S. Parameters plotted against values of molecular weights obtained for each protein, belonging to "mainly-beta" class. (A) MM-type H-bonds (B) total accessibility (C) void number (D) water molecules. [file 1472-6807-7-9-S1.pdf]

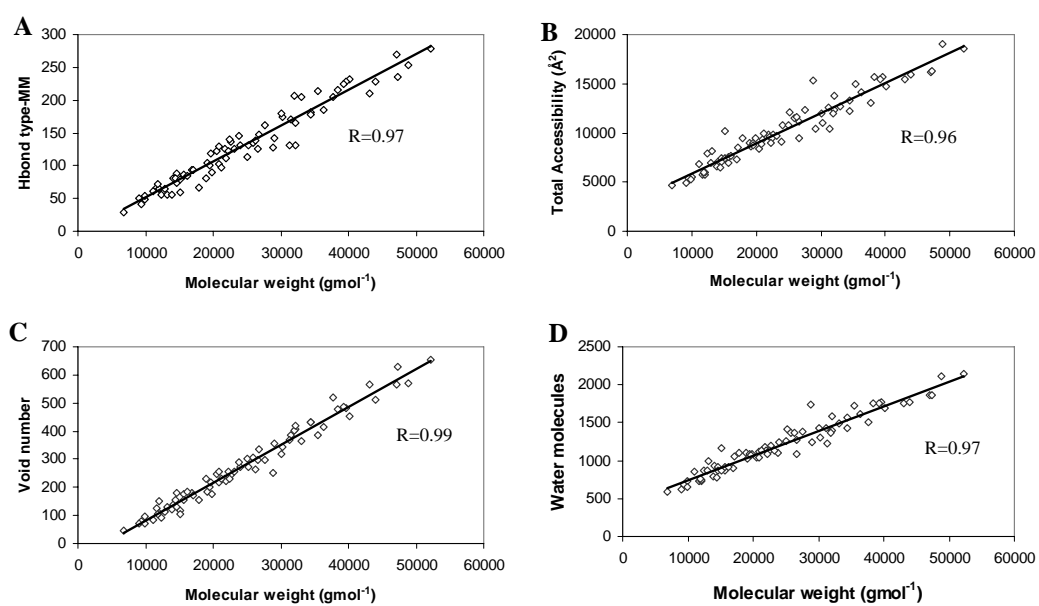

Figure2S

Supplement: Additional File 2 — Figure2S. Some parameters plotted against values of molecular weights obtained for each protein, belonging to "alpha/beta" class. (A) MM-type H-bonds (B) total accessibility (C) void number (D) water molecules. [file 1472-6807-7-9-S2.pdf]

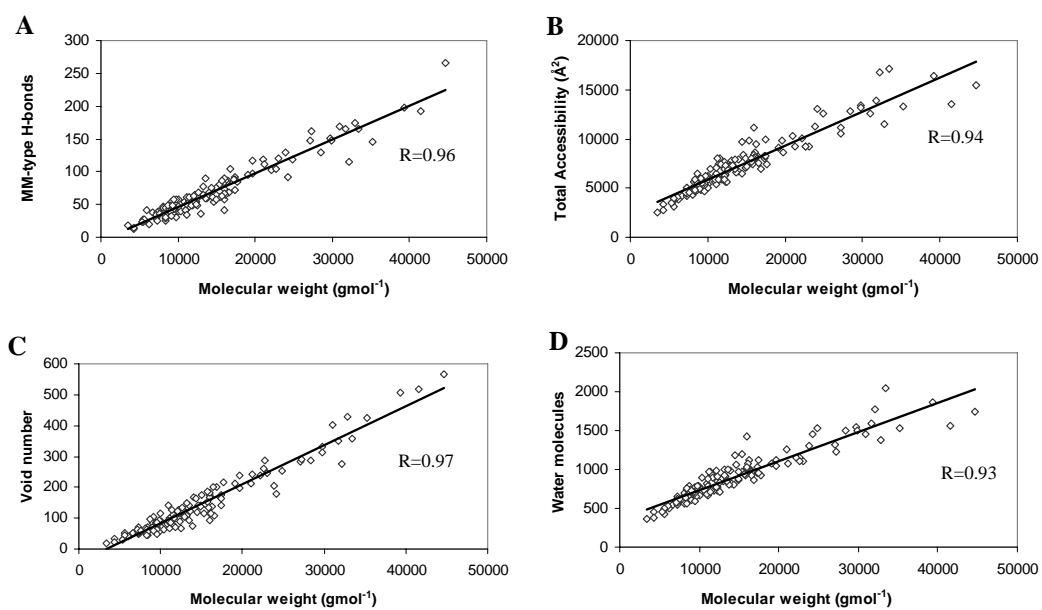

**Figure3S**

Supplement: Additional File 3 — Figure3S. Some parameters plotted against values of molecular weights obtained for each protein, belonging to "alpha+beta" class. (A) MM-type H-bonds (B) total accessibility (C) void number (D) water molecules. [file 1472-6807-7-9-S3.pdf]

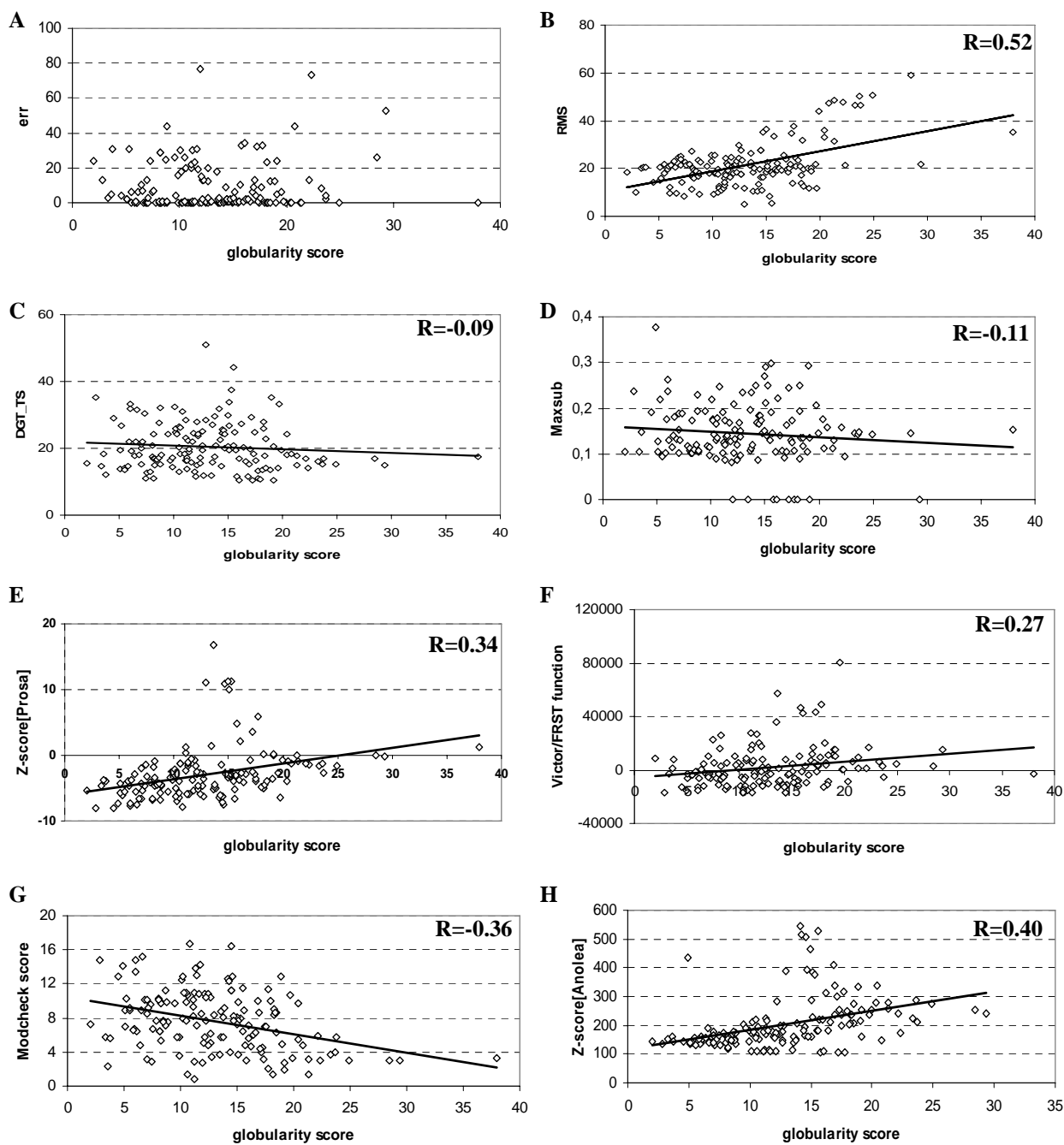

Figure4S.pdf

Supplement: Additional File 6 — Figure4S. Model evaluation parameters plotted against values of globularity score obtained for T0198 target (A) gross violations of distance constraints (err) (B) root-mean square deviations (RMSD) (C) Global Distance Test_Total Score (GDT_TS) (D) MaxSub score (E) PROSA II Z-score (F) Victor/FRST function (G) Modcheck score (H) Anolea Z-score. [file 1472-6807-7-9-S6.pdf]

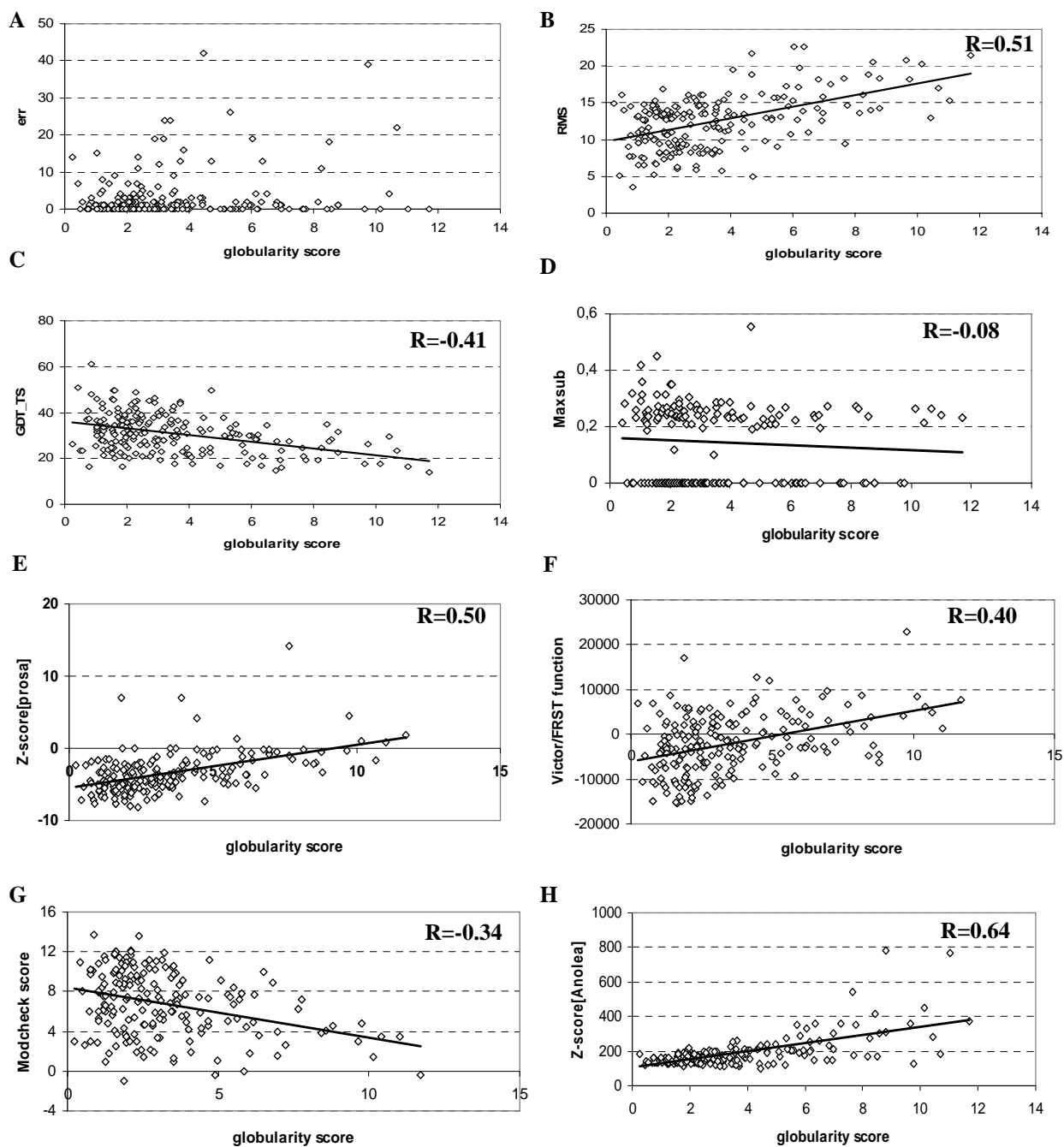

**Figure5S**

Supplement: Additional File 7 — Figure5S. Model evaluation parameters plotted against values of globularity score obtained for T0201 target (A) gross violations of distance constraints (err) (B) root-mean square deviations (RMSD) (C) Global Distance Test_Total Score (GDT_TS) (D) MaxSub score (E) PROSA II Z-score (F) Victor/FRST function (G) Modcheck score (H) Anolea Z-score. [file 1472-6807-7-9-S7.pdf]

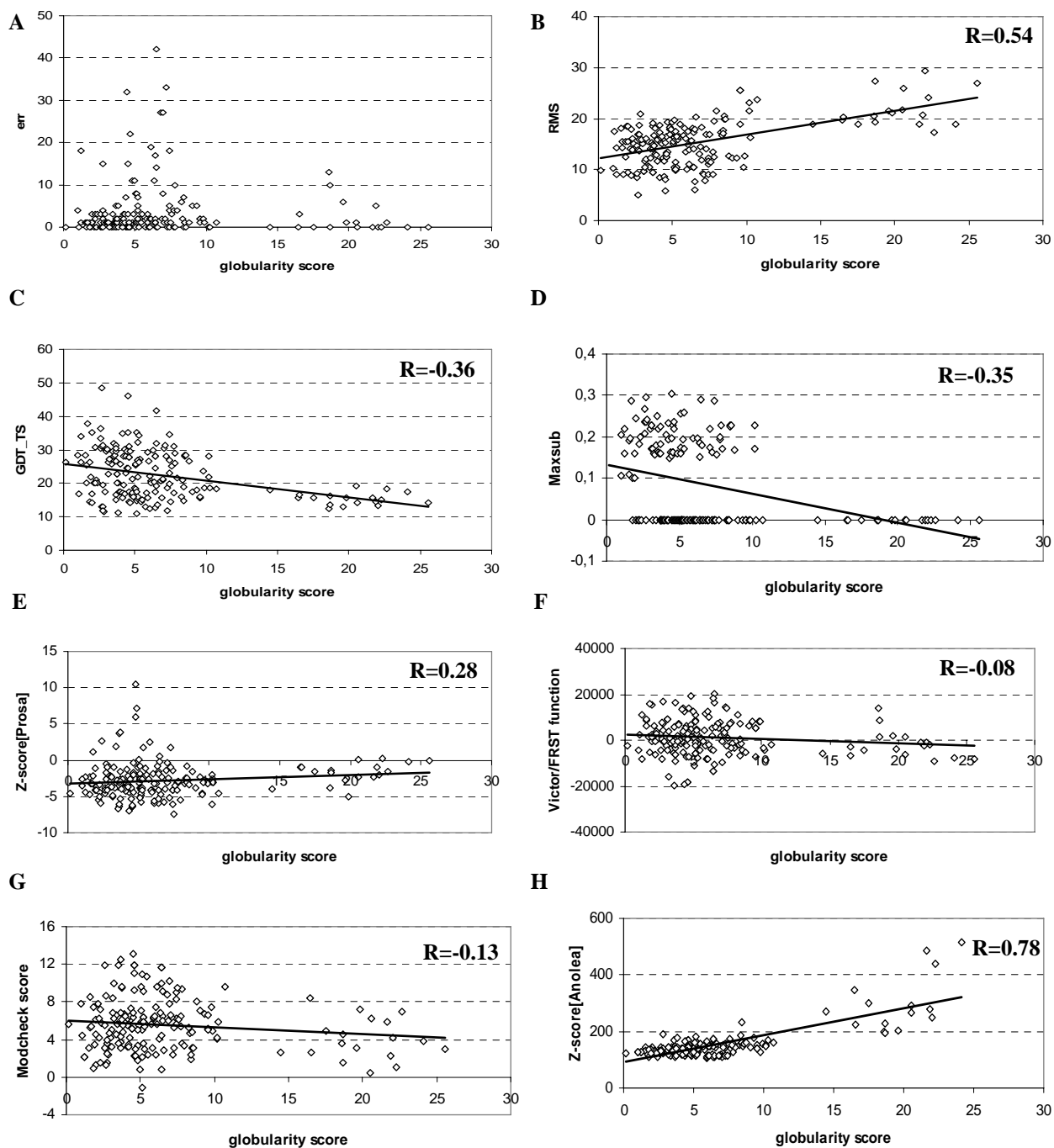

**Figure6S**

Supplement: Additional File 8 — Figure6S. Model evaluation parameters plotted against values of globularity score obtained for T0212 target (A) gross violations of distance constraints (err) (B) root-mean square deviations (RMSD) (C) Global Distance Test_Total Score (GDT_TS) (D) MaxSub score (E) PROSA II Z-score (F) Victor/FRST function (G) Modcheck score (H) Anolea Z-score. [file 1472-6807-7-9-S8.pdf]

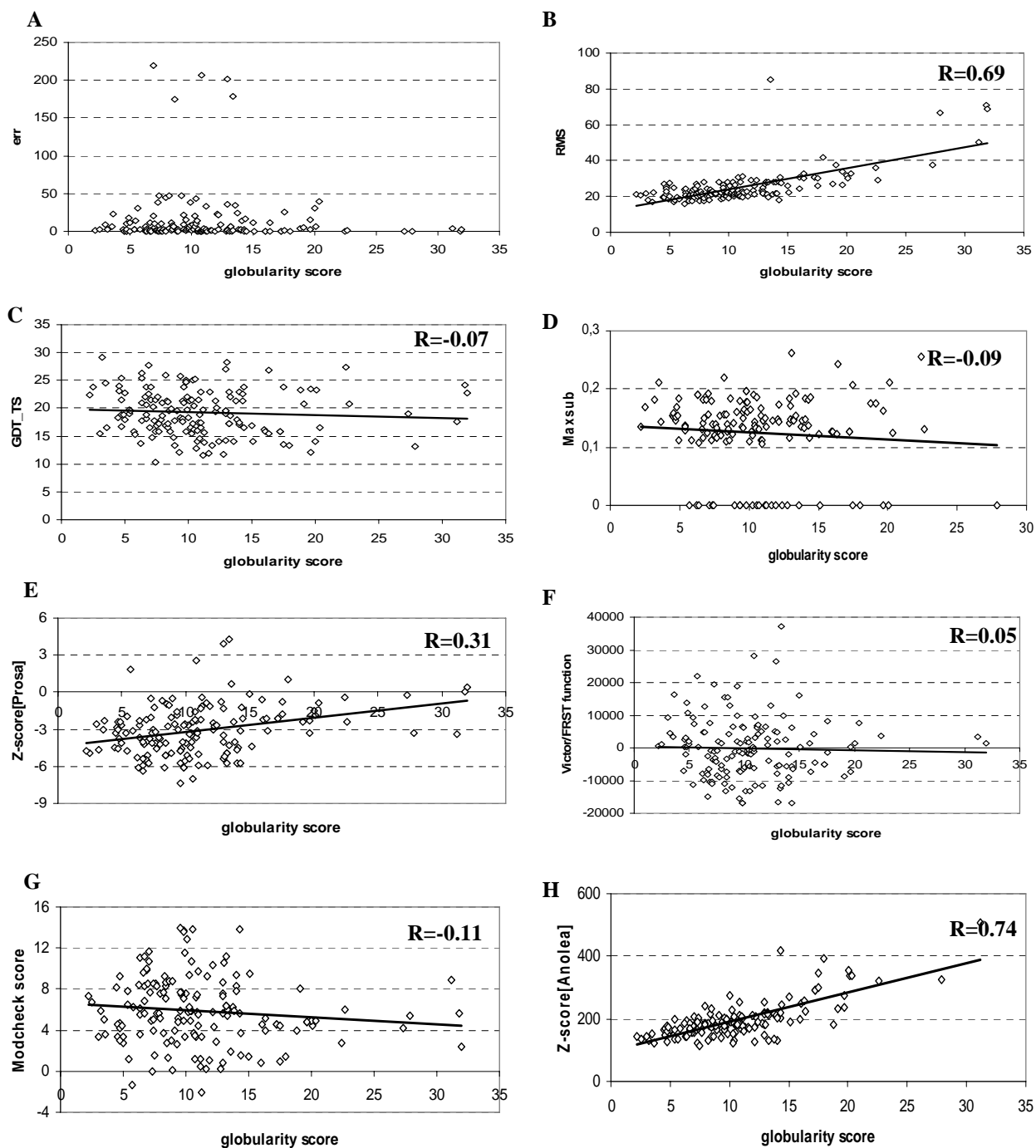

**Figure7S**

Supplement: Additional File 9 — Figure7S. Model evaluation parameters plotted against values of globularity score obtained for T0238 target (A) gross violations of distance constraints (err) (B) root-mean square deviations (RMSD) (C) Global Distance Test_Total Score (GDT_TS) (D) MaxSub score (E) PROSA II Z-score (F) Victor/FRST function (G) Modcheck score (H) Anolea Z-score. [file 1472-6807-7-9-S9.pdf]

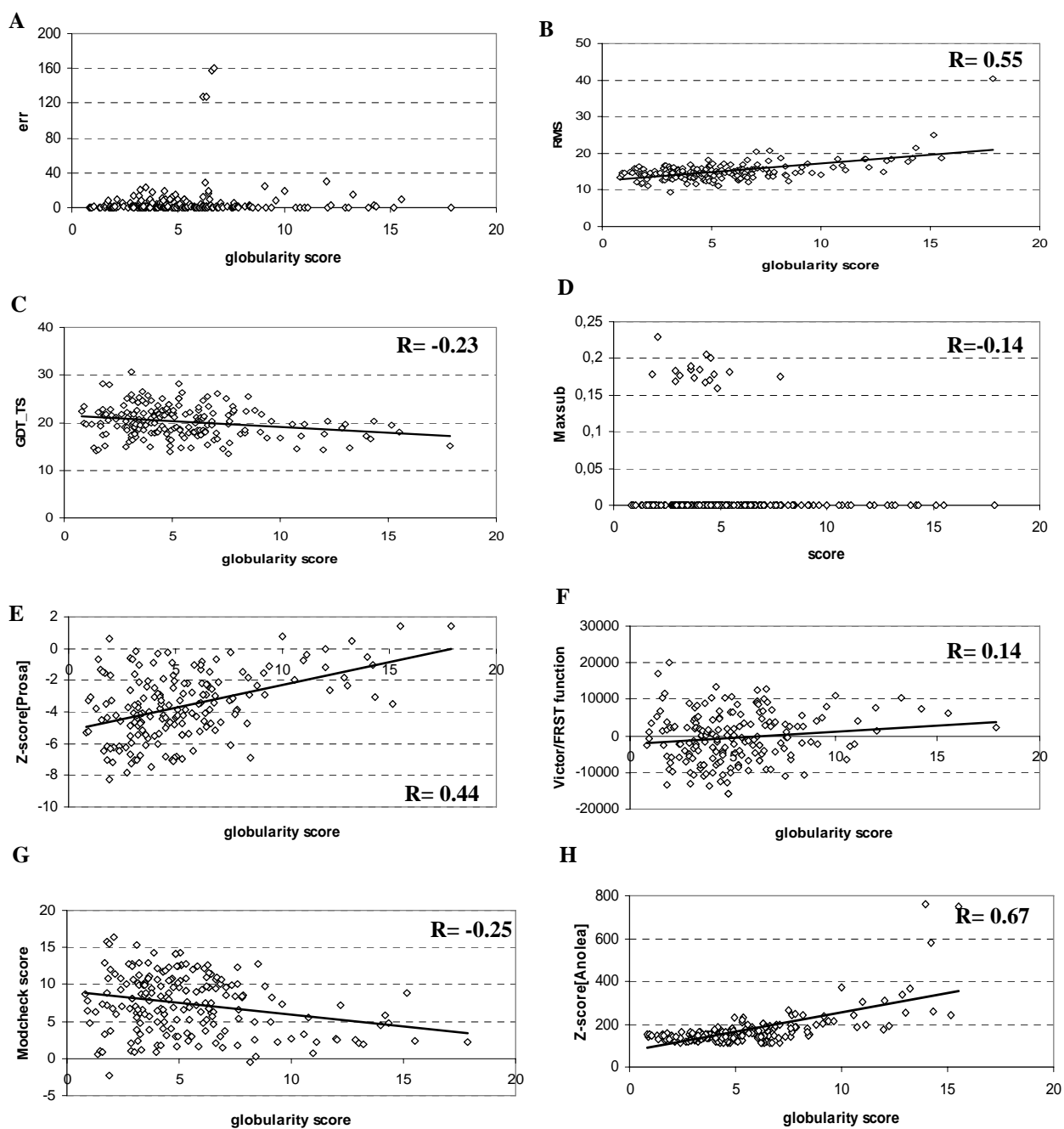

**Figure8S**

Supplement: Additional File 10 — Figure8S. Model evaluation parameters plotted against values of globularity score obtained for T0242 target (A) gross violations of distance constraints (err) (B) root-mean square deviations (RMSD) (C) Global Distance Test_Total Score (GDT_TS) (D) MaxSub score (E) PROSA II Z-score (F) Victor/FRST function (G) Modcheck score (H) Anolea Z-score. [file 1472-6807-7-9-S10.pdf]

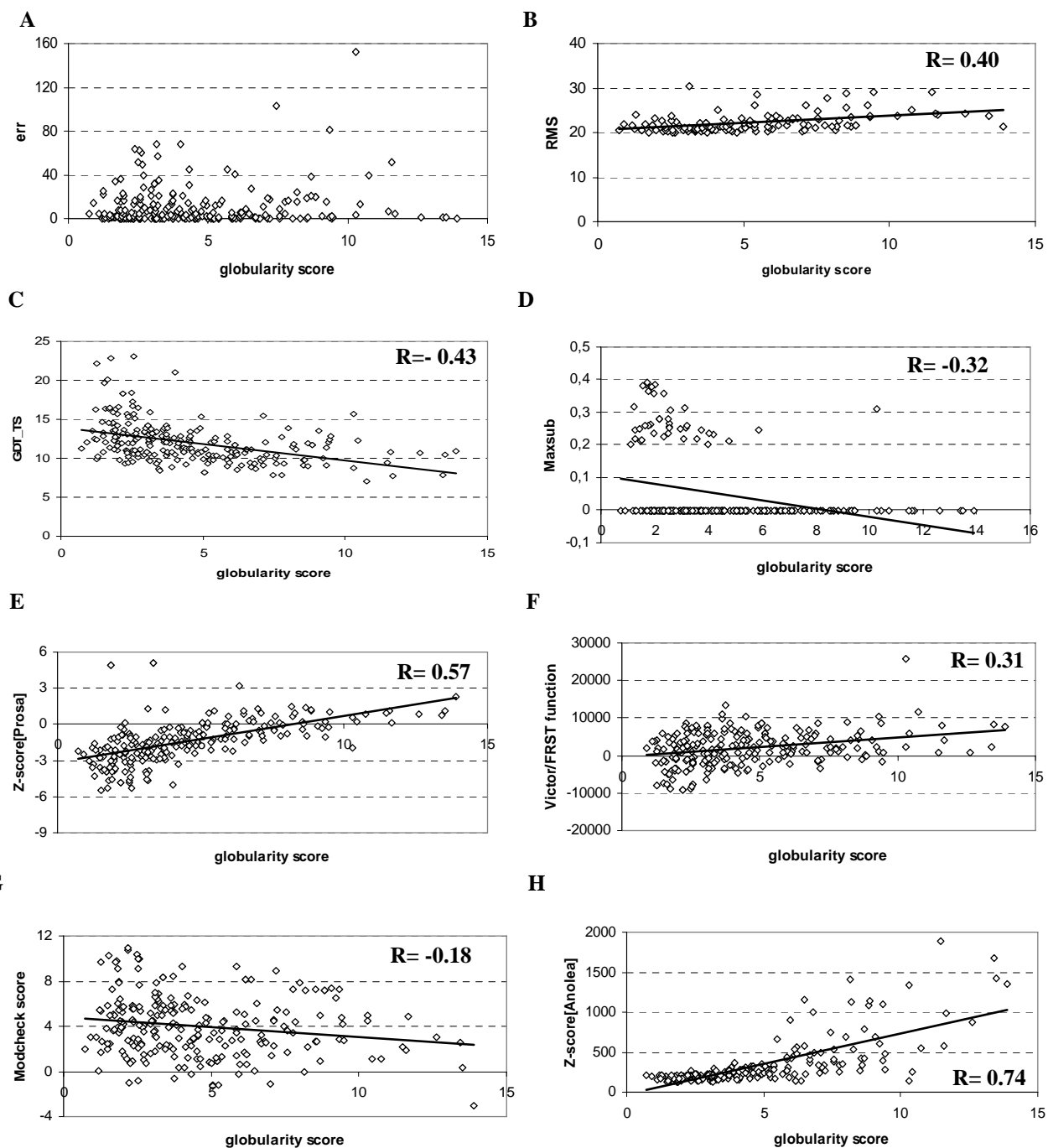

**Figure9S**

Supplement: Additional File 11 — Figure9S. Model evaluation parameters plotted against values of globularity score obtained for T0248 target (A) gross violations of distance constraints (err) (B) root-mean square deviations (RMSD) (C) Global Distance Test_Total Score (GDT_TS) (D) MaxSub score (E) PROSA II Z-score (F) Victor/FRST function (G) Modcheck score (H) Anolea Z-score. [file 1472-6807-7-9-S11.pdf]

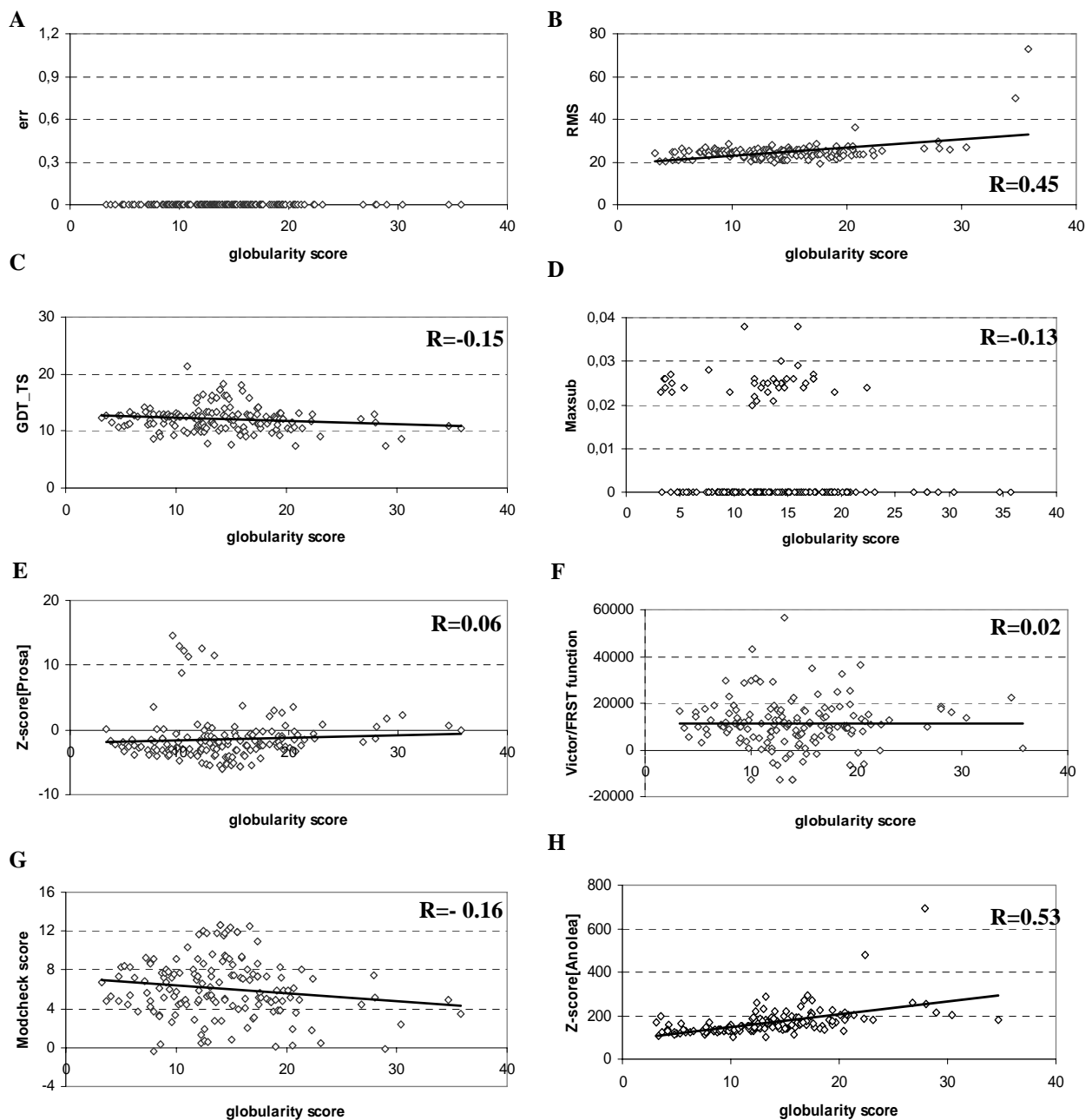

**Figure10S**

Supplement: Additional File 12 — Figure10S. Model evaluation parameters plotted against values of globularity score obtained for T0216_1 target (A) gross violations of distance constraints (err) (B) root-mean square deviations (RMSD) (C) Global Distance Test_Total Score (GDT_TS) (D) MaxSub score (E) PROSA II Z-score (F) Victor/FRST function (G) Modcheck score (H) Anolea Z-score. [file 1472-6807-7-9-S12.pdf]

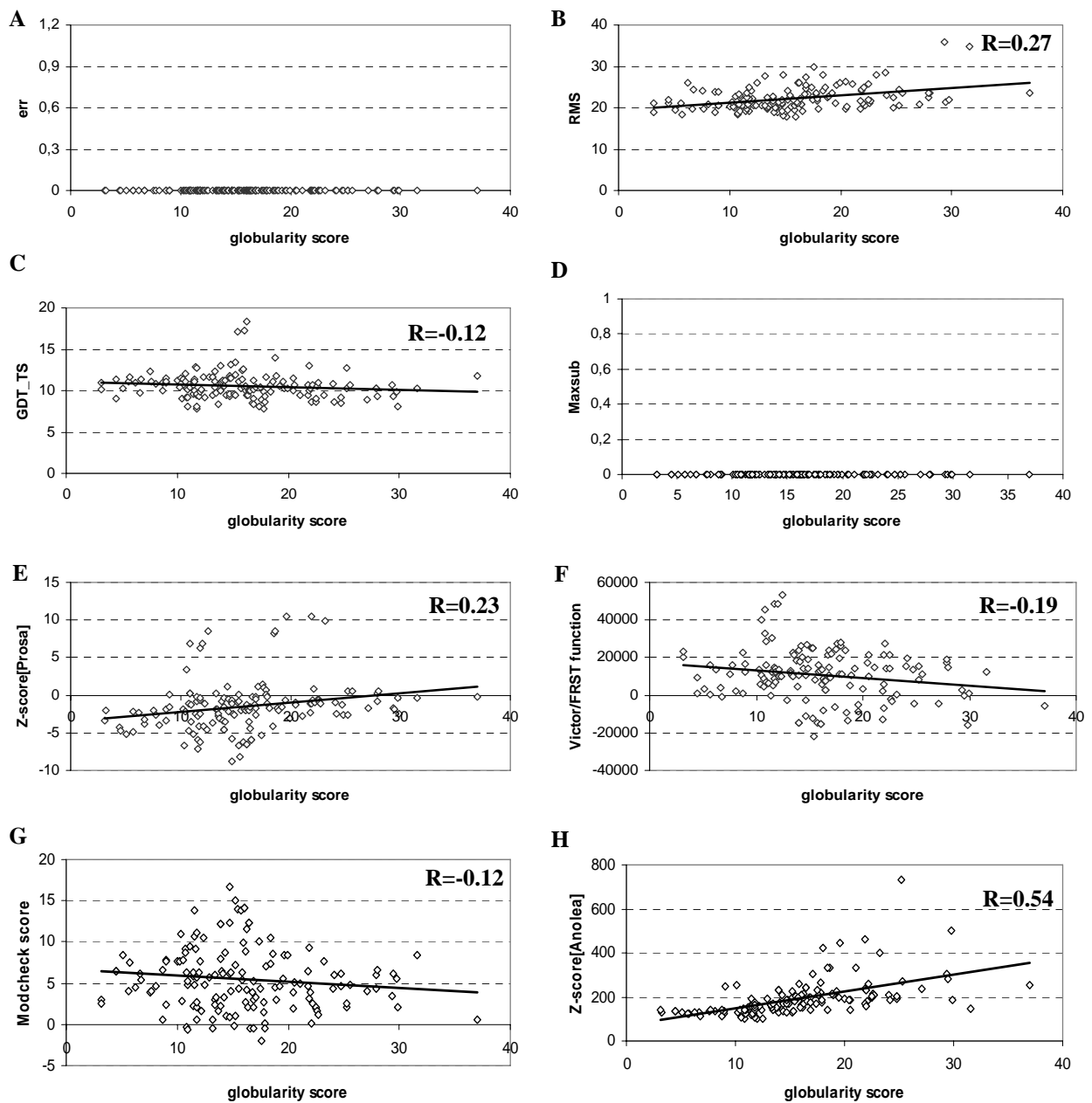

**Figure11S**

Supplement: Additional File 13 — Figure11S. Model evaluation parameters plotted against values of globularity score obtained for T0216_2 target (A) gross violations of distance constraints (err) (B) root-mean square deviations (RMSD) (C) Global Distance Test_Total Score (GDT_TS) (D) MaxSub score (E) PROSA II Z-score (F) Victor/FRST function (G) Modcheck score (H) Anolea Z-score. [file 1472-6807-7-9-S13.pdf]

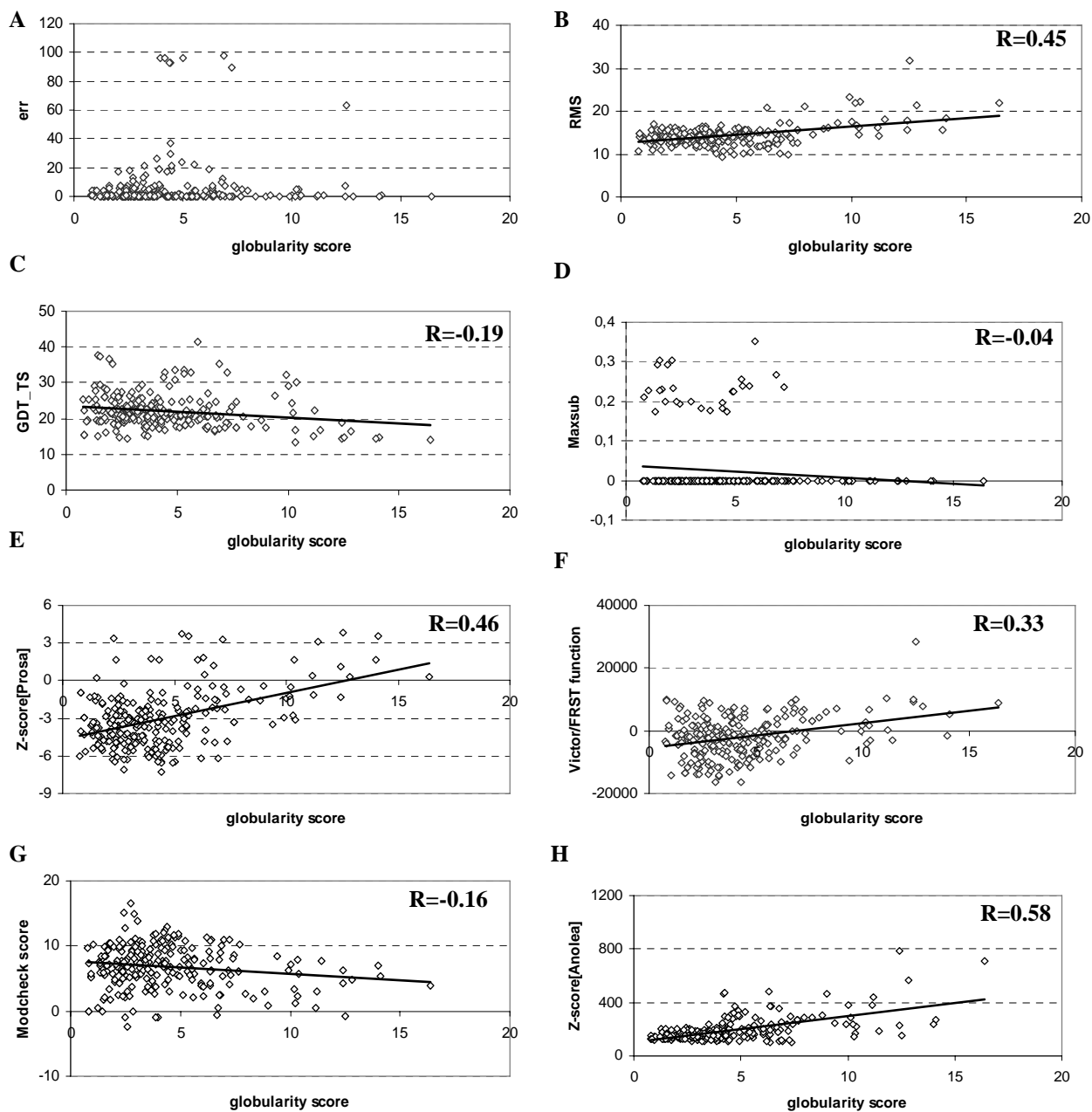

**Figure12S**

Supplement: Additional File 14 — Figure12S. Model evaluation parameters plotted against values of globularity score obtained for T0239 target (A) gross violations of distance constraints (err) (B) root-mean square deviations (RMSD) (C) Global Distance Test_Total Score (GDT_TS) (D) MaxSub score (E) PROSA II Z-score (F) Victor/FRST function (G) Modcheck score (H) Anolea Z-score. [file 1472-6807-7-9-S14.pdf]

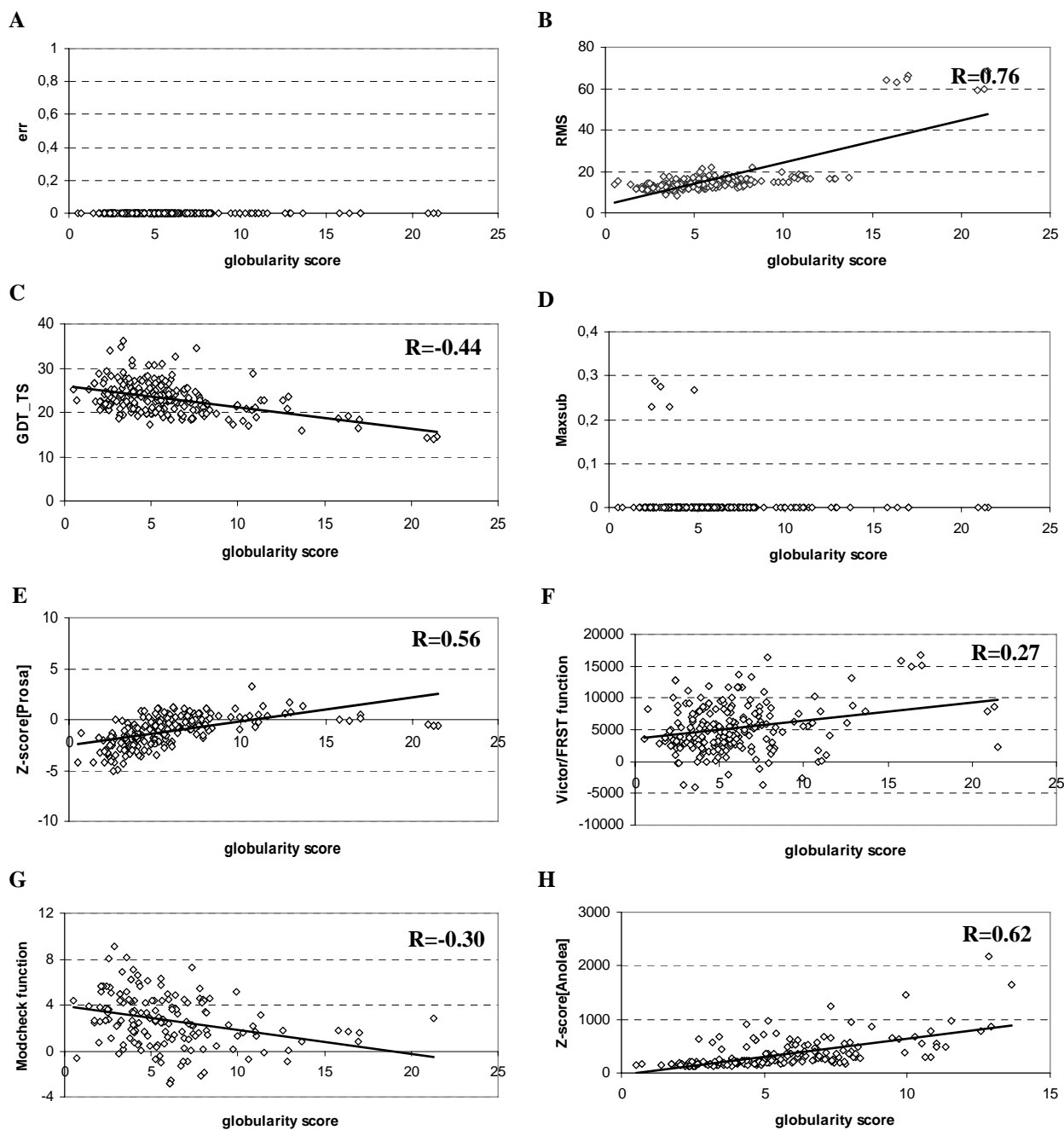

**Figure13S**

Supplement: Additional File 15 — Figure13S. Model evaluation parameters plotted against values of globularity score obtained for T0199_3 target (A) gross violations of distance constraints (err) (B) root-mean square deviations (RMSD) (C) Global Distance Test_Total Score (GDT_TS) (D) MaxSub score (E) PROSA II Z-score (F) Victor/FRST function (G) Modcheck score (H) Anolea Z-score. [file 1472-6807-7-9-S15.pdf]

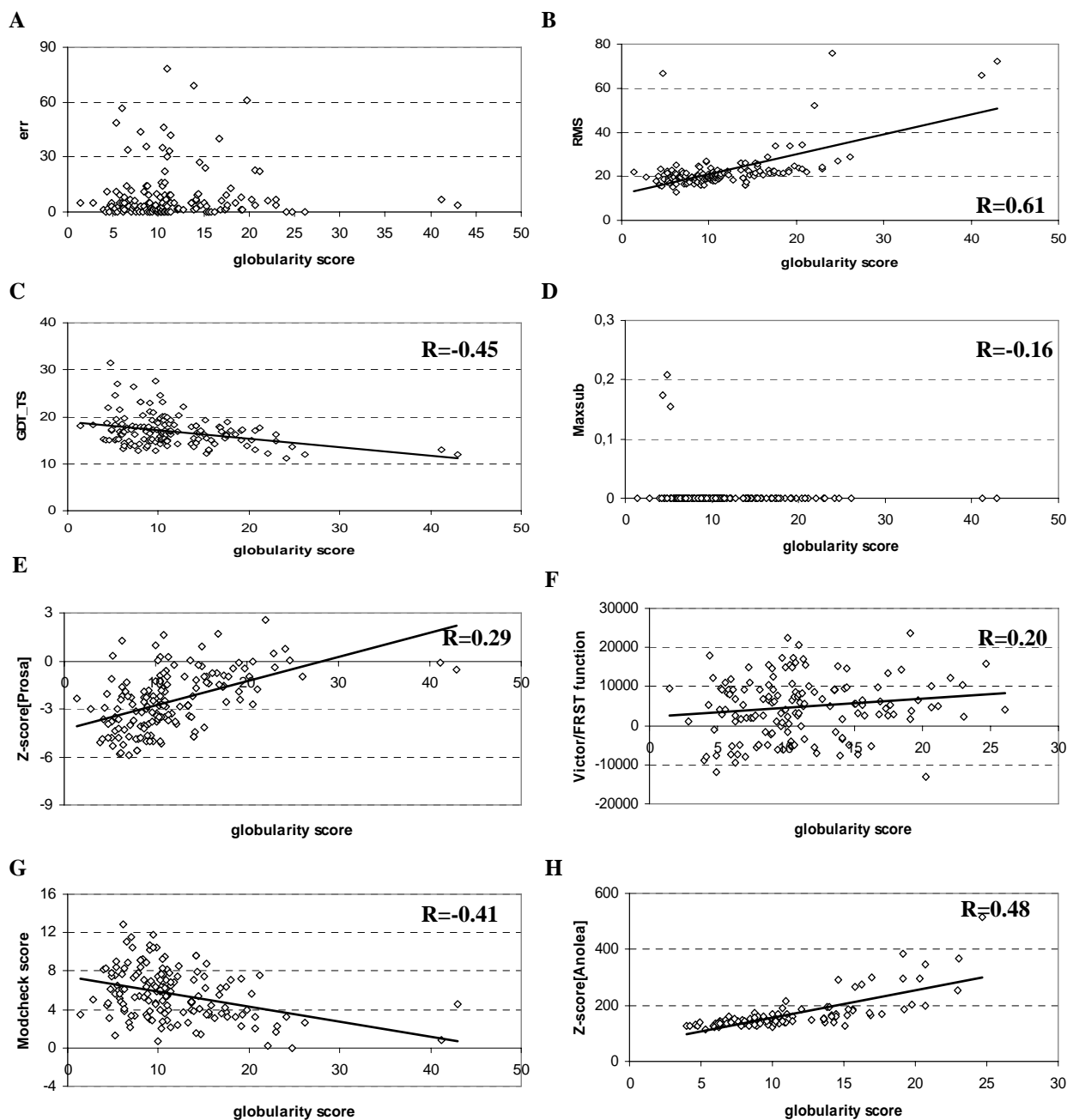

**Figure14S**

Supplement: Additional File 16 — Figure14S. Model evaluation parameters plotted against values of globularity score obtained for T0209_1 target (A) gross violations of distance constraints (err) (B) root-mean square deviations (RMSD) (C) Global Distance Test_Total Score (GDT_TS) (D) MaxSub score (E) PROSA II Z-score (F) Victor/FRST function (G) Modcheck score (H) Anolea Z-score. [file 1472-6807-7-9-S16.pdf]

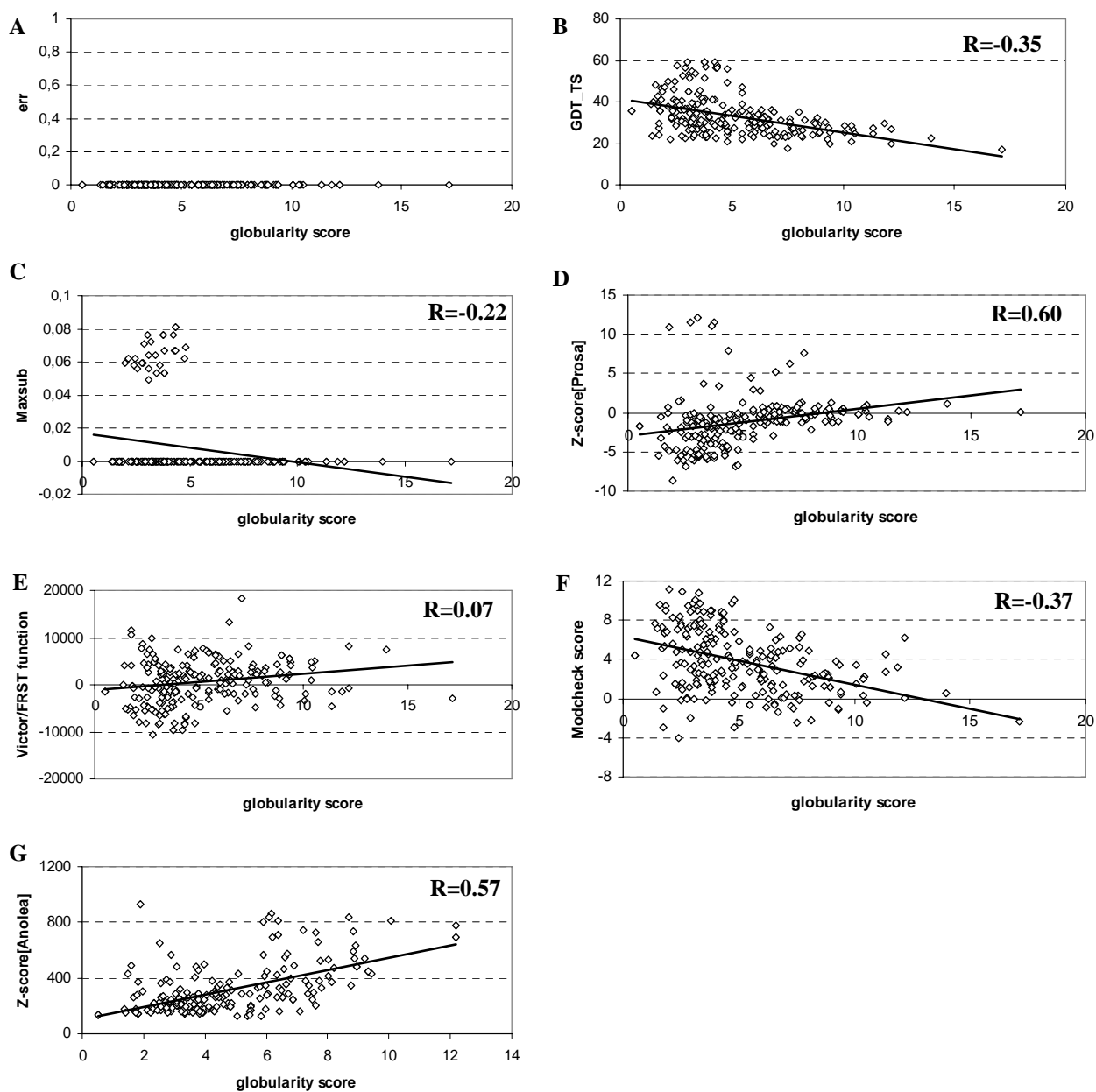

**Figure15S**

Supplement: Additional File 17 — Figure15S. Model evaluation parameters plotted against values of globularity score obtained for T0209_2 target (A) gross violations of distance constraints (err) (B) root-mean square deviations (RMSD) (C) Global Distance Test_Total Score (GDT_TS) (D) MaxSub score (E) PROSA II Z-score (F) Victor/FRST function (G) Modcheck score (H) Anolea Z-score. [file 1472-6807-7-9-S17.pdf]

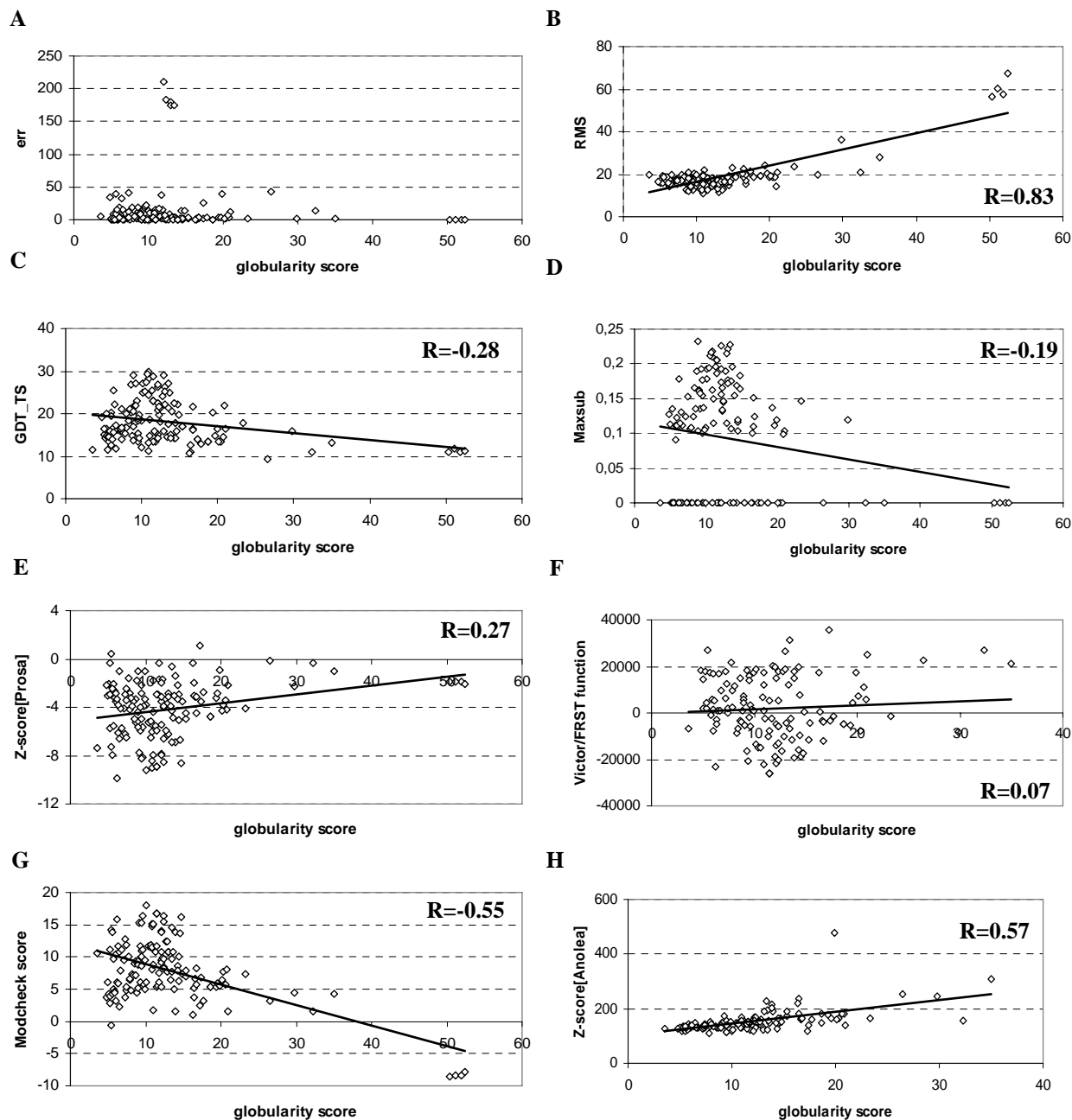

**Figure16S**

Supplement: Additional File 18 — Figure16S. Model evaluation parameters plotted against values of globularity score obtained for T0273 target (A) gross violations of distance constraints (err) (B) root-mean square deviations (RMSD) (C) Global Distance Test_Total Score (GDT_TS) (D) MaxSub score (E) PROSA II Z-score (F) Victor/FRST function (G) Modcheck score (H) Anolea Z-score. [file 1472-6807-7-9-S18.pdf]

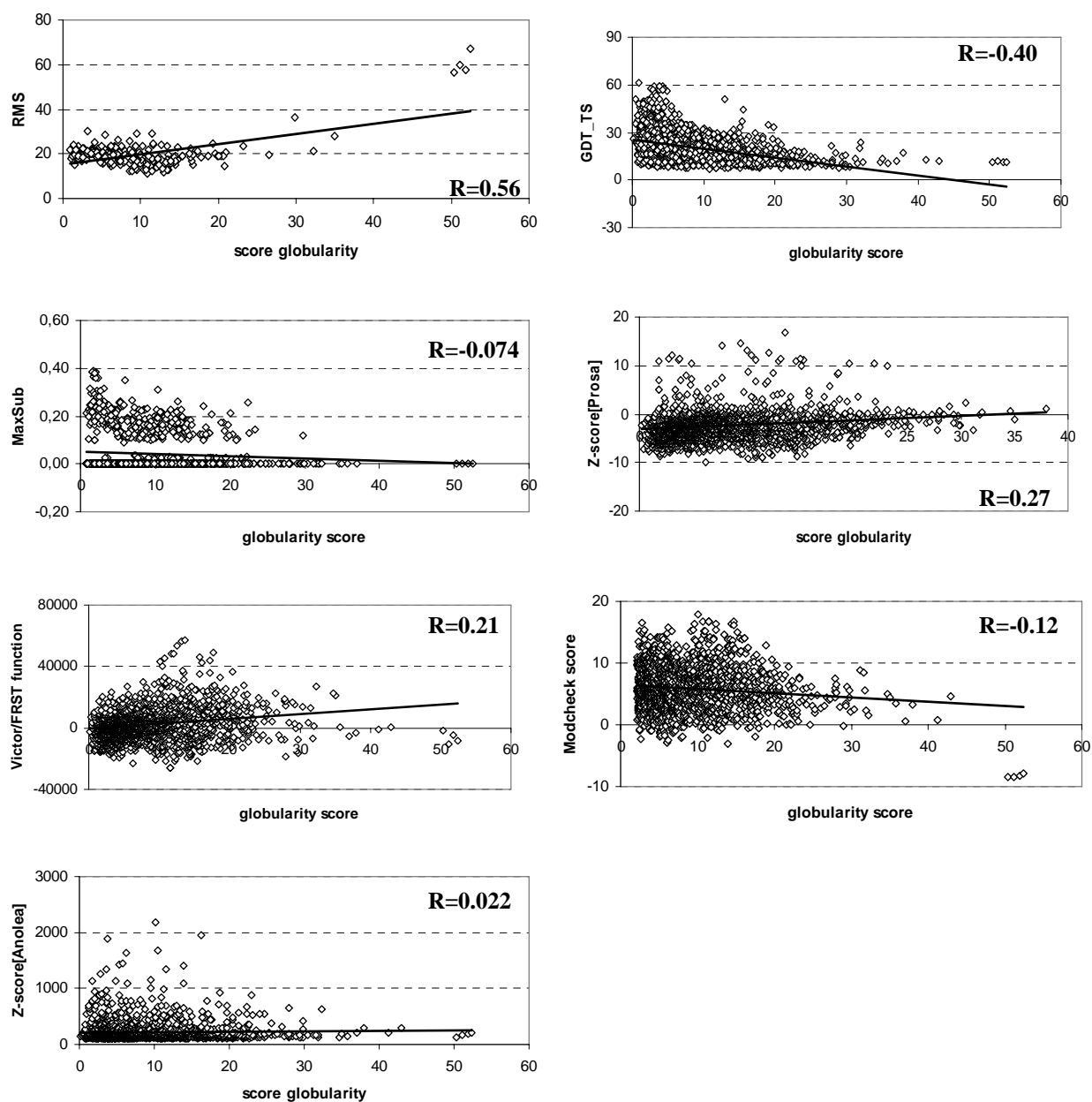

**Figure17S**

Supplement: Additional File 19 — Figure17S. Model evaluation parameters plotted against values of globularity score obtained for all models (A) gross violations of distance constraints (err) (B) root-mean square deviations (RMSD) (C) Global Distance Test_Total Score (GDT_TS) (D) MaxSub score (E) PROSA II Z-score (F) Victor/FRST function (G) Modcheck score (H) Anolea Z-score. [file 1472-6807-7-9-S19.pdf]
