# Supplementary material for: Evaluation of the structural quality of modeled proteins by using globularity criteria
Source: BMC Struct Biol. 2007 Mar 9;7:9. doi: 10.1186/1472-6807-7-9 (PMC1828058; doi:10.1186/1472-6807-7-9)
Supplement: Additional File 4 — Table1S. Theoretical parameters obtained analysing protein structures belonging to four structural classes. [file 1472-6807-7-9-S4.pdf]

Table1S

Theoretical parameters obtained analysing protein structures, belonging to four structural classes.

	Linear regression	RMSE
**mainly-alpha**		
**MM-type H-bonds**	y=0.0062x-6.5752	12.8
**Total Accessibility**	y=0.4093x+1904.1	851.7 ($Å^2$)
**Void number**	y=0.0102x-28.33	15.9
**Water number**	y=0.0444x+310.71	109.9
**mainly-beta**		
**MM-type H-bonds**	y=0.0039x-5.0753	10.3
**Total Accessibility**	y=0.4543+1310.5	735.5 ($Å^2$)
**Void number**	y=0.0109x-19.925	17.04
**Water number**	y=0.0499+234.99	92
**alpha/beta**		
**MM-type H-bonds**	y=0.0055x-3.6344	14.2
**Total Accessibility**	y=0.3044x+2876.8	898.5 ($Å^2$)
**Void number**	y=0.0135x-53.52	24.7
**Water number**	y=0.0326x+417	93
**alpha+beta**		
**MM-type H-bonds**	y=0.0051x-5.3549	11.6
**Total Accessibility**	y=0.3481x+2356.2	979.7 ($Å^2$)
**Void number**	y=0.0127x-44.115	25.6
**Water number**	y=0.0375x+360.99	114.7

[a]Linear regression equations are obtained reporting MM-type H-bonds, total Accessibility, void number and water molecules versus molecular weights (Fig. 1, 1S, 2S and 3S) and related Root Mean Square Errors (RMSE) calculated on the basis of predicted and calculated values (see Material and Methods). For RMSE related to total accessibility is reported the unit of measure in parentheses.
