# Supplementary material for: Evaluation of the structural quality of modeled proteins by using globularity criteria
Source: BMC Struct Biol. 2007 Mar 9;7:9. doi: 10.1186/1472-6807-7-9 (PMC1828058; doi:10.1186/1472-6807-7-9)
Supplement: Additional File 5 — Table2S. We have reported for each target and for the whole model set the cutoff value of globularity score, and the average values of RMSD and GDT_TS for the models above and below the cutoff. In parenthesis are reported the standard deviations. [file 1472-6807-7-9-S5.pdf]

Table2S

We have reported for each target and for the whole model set the cutoff value of globularity score, and the average values of RMSD and GDT_TS for the models above and below the cutoff. In parenthesis are reported the standard deviations.

**Target**	**cutoff**	**Average RMSD (below cutoff)**	**Average RMSD (above cutoff)**	**Average GDT_TS (below cutoff)**	**Average GDT_TS (above cutoff)**
**T0198**	5.9	13.9(3.5)	21.7(9.8)	28.9(7.4)	20.9(7.0)
**T0199_3**	5.1	12.9(1.7)	17.5(10)	24(3.5)	19.3(3.4)
**T0201**	5.1	11.5(3.2)	15.6(3.5)	32.5(5.2)	25.3(6.2)
**T0209_1**	5.9	18.6(3.7)	21.6(6.5)	34.2(5.6)	16.7(2.8)
**T0209_2**	5.1	N/A	N/A	37.1(5.3)	25.2(5.2)
**T0212**	5.9	14.2(3.4)	16.7(5.2)	23.8(7.6)	10.8(6.3)
**T0216_1**	5.1	11.3(2.3)	24.7(4.9)	21.7(2.1)	12.1(2.2)
**T0216_2**	5.1	10.2(1.3)	23.3(12.2)	24.3(3.2)	10.6(1.6)
**T0238**	5.9	15.3(3.6)	25.2(9.8)	24.7(5.0)	19.2(4.1)
**T0239**	5.1	13.8(1.3)	21.4(5.5)	22.3(4.1)	15.3(3.5)
**T0242**	5.1	14.3(1.4)	19.6(3.2)	20.7(3.2)	15.7(3.5)
**T0248**	5.1	12.7(2.7)	22.5(4.2)	19.9(1.8)	10.6(1.6)
**T0273**	5.1	15.6(3.2)	23.6(7.9)	17.2(1.9)	13.2(4.9)
**All models**		15.1(4.4)	22.8(8.9)	24.1(9.7)	15.2(6.6)
