# Supplementary material for: Evaluation of the structural quality of modeled proteins by using globularity criteria
Source: BMC Struct Biol. 2007 Mar 9;7:9. doi: 10.1186/1472-6807-7-9 (PMC1828058; doi:10.1186/1472-6807-7-9)
Supplement: Additional File 20 — Table3S. Correlation coefficients between all methods, as well as for our four individual features (MM-type H-bonds, void number, water molecules and total accessibility), and three correct quality measures (i.e. RMSD, GDT_TS and MaxSub) evaluated for each of 13 target. [file 1472-6807-7-9-S20.pdf]

Table 3S

Correlation coefficients between all quality assessment methods (Prosa, Modcheck, Victor/FRST, Anolea and Globularity score), as well as our four individual features (MM-type H-bonds, void number, water molecules and total accessibility), and three correct quality measures (i.e. RMSD, GDT_TS and MaxSub) evaluated for each of 13 target.

	RMS	GDT_TS	MaxSub
**T0198**			
**Z-score [Prosa]**	0.14	-0.19	-0.17
**Modcheck score**	-0.42	0.57	0.45
**Victor/FRST function**	0.07	-0.45	-0.5
**Z-score [Anolea]**	0.02	0.42	0.48
**Globularity score**	0.52	-0.07	-0.053
**MM H-bond**	0.002	0.57	0.66
**Void number**	-0.1	-0.29	-0.28
**Total Accessibility**	0.4	-0.32	-0.12
**Water molecules**	0.71	-0.22	-0.008
**T0199_3**			
**Z-score [Prosa]**	0.04	0.04	-0.05
**Modcheck score**	-0.17	0.24	0.09
**Victor/FRST function**	0.23	-0.2	-0.07
**Z-score [Anolea]**	0.43	-0.18	-0.04
**Globularity score**	0.69	-0.38	-0.11
**MM H-bond**	-0.13	0.47	0.18
**Void number**	-0.34	0.1	-0.08
**Total Accessibility**	0.64	-0.28	-0.12
**Water molecules**	0.74	-0.29	-0.13
**T0201**			
**Z-score [Prosa]**	0.38	-0.54	-0.43
**Modcheck score**	-0.52	0.69	0.61

**Victor/FRST function**	0.37	-0.54	-0.47
**Z-score [Anolea]**	0.46	-0.32	-0.16
**Globularity score**	0.51	-0.4	-0.24
**MM H-bond**	0.2	0.43	0.3
**Void number**	-0.17	-0.026	-0.07
**Total Accessibility**	0.52	-0.38	-0.24
**Water molecules**	0.58	-0.41	-0.26

**T0209_1**			
**Z-score [Prosa]**	0.33	-0.41	-0.12
**Modcheck score**	-0.32	0.37	-0.015
**Victor/FRST function**	-0.029	-0.036	-0.0098
**Z-score [Anolea]**	0.68	-0.24	-0.053
**Globularity score**	0.61	-0.34	-0.16
**MM H-bond**	-0.1	0.15	0.04
**Void number**	-0.38	0.19	0.12
**Total Accessibility**	0.57	-0.33	-0.16
**Water molecules**	0.69	-0.35	-0.14

**T0209_2**			
**Z-score [Prosa]**	N/A	0.078	0.14
**Modcheck score**	N/A	0.73	0.47
**Victor/FRST function**	N/A	-0.52	-0.42
**Z-score [Anolea]**	N/A	-0.14	-0.16
**Globularity score**	N/A	-0.45	-0.22
**MM H-bond**	N/A	0.53	0.32
**Void number**	N/A	0.017	0.16
**Total Accessibility**	N/A	-0.39	-0.16
**Water molecules**	N/A	-0.46	-0.21

T0212

**Z-score [Prosa]**	-0.17	-0.18	-0.17
**Modcheck score**	-0.37	0.23	0.013
**Victor/FRST function**	0.07	-0.26	-0.18
**Z-score [Anolea]**	0.37	-0.25	-0.21
**Globularity score**	0.54	-0.36	-0.34
**MM H-bond**	-0.2	0.09	0.15
**Void number**	-0.33	0.2	0.21
**Total Accessibility**	0.47	-0.23	-0.2
**Water molecules**	0.54	-0.24	-0.19
**T0216_1**			
**Z-score [Prosa]**	0.08	-0.29	0.07
**Modcheck score**	-0.16	0.7	0.045
**Victor/FRST function**	-0.019	-0.4	0.046
**Z-score [Anolea]**	0.16	0.013	-0.058
**Globularity score**	0.45	-0.15	-0.16
**MM H-bond**	0.17	0.25	0.026
**Void number**	-0.23	-0.0019	0.12
**Total Accessibility**	0.34	-0.0019	-0.12
**Water molecules**	0.49	-0.0074	-0.12
**T0216_2**			
**Z-score [Prosa]**	0.048	-0.29	N/A
**Modcheck score**	-0.084	0.44	N/A
**Victor/FRST function**	-0.066	-0.31	N/A
**Z-score [Anolea]**	0.05	-0.016	N/A
**Globularity score**	0.27	-0.12	N/A
**MM H-bond**	-0.049	0.24	N/A
**Void number**	-0.086	-0.13	N/A
**Total Accessibility**	0.32	-0.074	N/A
**Water molecules**	0.29	-0.1	N/A

T0238

**Z-score [Prosa]**	-0.083	-0.22	-0.34
**Modcheck score**	-0.015	0.15	0.4
**Victor/FRST function**	0.031	-0.23	-0.23
**Z-score [Anolea]**	0.036	-0.082	-0.13
**Globularity score**	-0.062	-0.076	-0.21
**MM H-bond**	-0.065	0.086	0.46
**Void number**	-0.014	0.043	-0.098
**Total Accessibility**	-0.041	-0.035	-0.11
**Water molecules**	-0.021	0.03	-0.12

T0239

**Z-score [Prosa]**	0.39	-0.24	0.023
**Modcheck score**	-0.37	0.32	0.059
**Victor/FRST function**	0.34	-0.26	0.005
**Z-score [Anolea]**	0.36	-0.22	-0.16
**Globularity score**	0.45	-0.19	-0.11
**MM H-bond**	-0.23	0.26	0.14
**Void number**	0.58	-0.24	-0.13
**Total Accessibility**	0.64	-0.22	-0.11
**Water molecules**	-0.045	-0.078	-0.022

T0242

**Z-score [Prosa]**	0.36	-0.56	-0.13
**Modcheck score**	-0.36	0.62	0.13
**Victor/FRST function**	0.15	-0.54	-0.19
**Z-score [Anolea]**	0.4	-0.17	-0.054
**Globularity score**	0.55	-0.23	-0.14
**MM H-bond**	0.07	0.085	0.098
**Void number**	-0.23	-0.056	0.0058

**Total Accessibility**	0.55	-0.21	-0.11
**Water molecules**	0.65	-0.25	-0.13
**T0248**			
**Z-score [Prosa]**	0.35	-0.61	-0.57
**Modcheck score**	-0.16	0.49	0.44
**Victor/FRST function**	0.07	-0.4	-0.41
**Z-score [Anolea]**	0.43	-0.3	-0.22
**Globularity score**	0.4	-0.43	-0.31
**MM H-bond**	-0.06	0.33	0.21
**Void number**	-0.24	0.2	0.17
**Total Accessibility**	0.43	0.51	-0.42
**Water molecules**	0.46	-0.53	0.42
**T0273**			
**Z-score [Prosa]**	0.24	-0.49	-0.4
**Modcheck score**	-0.14	0.54	0.53
**Victor/FRST function**	-0.09	-0.32	-0.37
**Z-score [Anolea]**	0.34	-0.15	-0.056
**Globularity score**	0.83	-0.28	-0.19
**MM H-bond**	-0.25	0.27	0.29
**Void number**	-0.53	0.086	0.042
**Total Accessibility**	0.8	-0.22	-0.12
**Water molecules**	0.89	-0.28	-0.18
