# Supplementary material for: Evaluation of the structural quality of modeled proteins by using globularity criteria
Source: BMC Struct Biol. 2007 Mar 9;7:9. doi: 10.1186/1472-6807-7-9 (PMC1828058; doi:10.1186/1472-6807-7-9)
Supplement: Additional File 21 — Table4S. A. For each target, columns report the number of models analyzed and the ratios of models for which the voids, MM-type H-bonds, water molecules, the total accessibility and the score values resulted within expected ranges for globular proteins and calculated as reported in Methods (see Additional File 4). B. For each target and for the whole set, columns report average GDT_TS value evaluated for models that have the number of void, MM-type H-bonds and water molecules, and the total accessibility within the expected range. In parenthesis are reported the standard deviations. [file 1472-6807-7-9-S21.pdf]

Table 4S

A. For each target, columns report the number of models analyzed and the ratios of models for which the voids, MM-type H-bonds, water molecules, the total accessibility and the score values resulted within expected ranges for globular proteins and calculated as reported in Methods (see Table 1S).

B. For each target and for the whole set, columns report average GDT_TS value evaluated for models that have the number of void, MM-type H-bonds and water molecules, and the total accessibility within the expected range. In parenthesis are reported the standard deviations.

A

	**Number of models**	**Number of models for which each single property is within the expected range**				
		**Void number**	**MM-type H-bonds**	**Water molecules**	**Total accessibility**	**score**
**T0198**	152	9/152	29/152	43/152	27/152	14/152
**T0238**	154	26/154	55/154	32/154	17/154	21/154
**T0209_1**	152	23/152	32/152	26/152	22/152	20/152
**T0212**	181	51/181	54/181	105/181	89/181	107/181
**T0199_3**	223	94/223	106/223	82/223	77/223	110/223
**T0201**	203	141/203	126/203	146/203	141/203	162/203
**T0209_2**	229	204/229	153/229	66/229	47/229	135/299
**T0216_1**	155	7/155	34/155	16/155	13/155	10/155
**T0216_2**	147	9/147	11/147	19/147	13/147	7/147
**T0239**	251	185/251	105/251	180/251	174/251	178/251
**T0248_2**	238	130/238	141/238	120/238	122/238	161/238
**T0242**	200	71/200	64/200	116/200	112/200	114/200
**T0273**	141	9/141	11/141	27/141	18/141	14/141

B

Average GDT_TS					
	**Void number**	**MM-type H-bonds**	**Water molecules**	**Total accessibility**	**Globulatity score**
**T0198**	22.6(8.5)	19.9(4.9)	22.3(8.7)	22.9(9.9)	28.9(7.4)
**T0238**	18.9(3.2)	20.1(3.7)	19.6(4.2)	19.0(3.9)	24.7(5.0)
**T0209_1**	26.7(3.9)	27.0(2.9)	27.8(4.3)	27.7(4.8)	34.2(5.6)
**T0212**	24.1(8.0)	24.5(8.1)	23.5(7.1)	23.4(6.9)	23.8(7.6)
**T0199_3**	23.4(3.1)	24.6(3.5)	24.1(3.6)	23.6(3.5)	24(3.5)
**T0201**	31.3(8.5)	32.4(8.6)	32.9(8.1)	32.8(8.3)	32.5(5.2)
**T0209_2**	33.1(9.0)	34.9(9.6)	36.7(9.5)	35.3(8.6)	37.1(5.3)
**T0216_1**	18.1(0.9)	18.0(1.9)	21.9(0.9)	17.1(0.9)	21.7(2.1)
**T0216_2**	19.8(1.2)	20.5(1.3)	20.5(0.8)	20.5(0.8)	24.3(3.2)
**T0239**	22.0(4.6)	22.8(4.5)	22.1(4.1)	22.2(4.1)	22.3(4.1)
**T0248_2**	17.4(2.8)	17.3(2.7)	18.1(2.9)	18.1(2.9)	19.9(1.8)
**T0242**	19.8(3.1)	20.4(3.4)	20.8(3.1)	20.8(3.0)	20.7(3.2)
**T0273**	16.4(3.6)	16.3(3.1)	16.3(3.1)	15.6(1.9	17.2(1.9)
**All models**	22.9(8.9)	22.5(9.3)	22.5(8.4)	22.4(8.2)	24.1(9.7)
