# Supplementary material for: Evaluation of the structural quality of modeled proteins by using globularity criteria
Source: BMC Struct Biol. 2007 Mar 9;7:9. doi: 10.1186/1472-6807-7-9 (PMC1828058; doi:10.1186/1472-6807-7-9)
Supplement: Additional File 22 — Table5S. Number of models for which the combination of two, three or four parameters (voids, MM-type H-bonds, water molecules and total accessibility) resulted in the expected ranges for globular proteins and calculated as reported in Methods (see Additional File 4). [file 1472-6807-7-9-S22.pdf]

Table5S

In the columns, we reported the number of models for which the combination of two, three or four parameters (voids, MM-type H-bonds, water molecules and total Accessibility) resulted in the expected ranges for globular proteins and calculated as reported in Methods (see Table 1S)

Target	Hbond void	Hbond Acc	Hbond water	void Acc	void water	Acc water	Hbond Acc void	Hbond acc water	Hbond void water	Acc void water	All four
**T0198**	2	2	6	2	3	23	1	2	1	2	1
**T0238**	5	3	6	6	9	15	0	3	1	6	0
**T0209_1**	6	7	6	9	9	19	4	7	4	7	4
**T0212**	24	34	41	33	37	85	19	30	19	29	17
**T0199_3**	45	45	48	31	35	69	20	42	22	28	20
**T0201**	96	99	106	113	117	140	81	98	84	110	78
**T0209_2**	138	34	47	33	55	47	25	32	42	32	25
**T0216_1**	0	6	7	0	0	12	0	6	0	0	0
**T01216_2**	0	3	6	2	3	13	0	3	0	2	0
**T0239**	76	80	82	124	138	149	64	72	68	118	59
**T0248**	81	80	79	86	83	116	56	77	56	89	67
**T0242**	30	44	41	52	48	106	27	40	23	44	23
**T0273**	0	0	3	6	6	16	0	0	0	5	0
